# Insights into *Helicobacter pylori* macrolide resistance: a comprehensive systematic review and meta-analysis

**DOI:** 10.3389/fmicb.2024.1481763

**Published:** 2024-10-30

**Authors:** Safoura Morad Kasani, Maryam Mofid, Tahereh Navidifar, Narges Golab, Elnaz Parvizi, Farzad Badmasti, Mohammad Sholeh, Masoumeh Beig

**Affiliations:** ^1^Department of Bacteriology, Pasteur Institute of Iran, Tehran, Iran; ^2^School of Medicine, Hamadan University of Medical Science, Hamadan, Iran; ^3^Department of Basic Sciences, Shoushtar Faculty of Medical Sciences, Shoushtar, Iran; ^4^Department of Microbiology, School of Medicine, Tehran University of Medical Sciences, Tehran, Iran; ^6^Student Research Committee, Pasteur Institute of Iran, Tehran, Iran; ^5^Department of Microbiology, Science and Research Branch, Islamic Azad University, Fars, Iran

**Keywords:** *Helicobacter pylori*, clarithromycin, erythromycin, azithromycin, antibiotic resistance, macrolides

## Abstract

**Background:**

*Helicobacter pylori* infection is a primary global health concern. However, the widespread use of antibiotics, particularly macrolides such as clarithromycin, has increased resistance among *H. pylori* strains. This study aimed to investigate the prevalence of macrolide resistance in *H. pylori* in different world regions.

**Methods:**

This systematic literature search was performed using the appropriate search syntax after searching PubMed, Embase, Web of Science, and Scopus databases between May 2015 and December 2023. Statistical analysis was performed using Pooled and random effects model in *R* and the metafor package.

**Results:**

A total of 7,768 articles were retrieved. After a thorough evaluation, 155 studies (by 178 reports) were finally eligible for inclusion in this systematic review and meta-analysis. According to the results, the majority of studies (178 reports from 43 countries) assessed clarithromycin susceptibility, with a pooled prevalence of 33.3% and high heterogeneity between studies (*I*^2^ = 98.57%, *p* < 0.001). The rate of erythromycin resistance was moderate (22.8%, 10 reports), while azithromycin resistance was 34.4% (4 reports). Subgroup analysis revealed significant differences in the prevalence of resistance based on geographic location, continent, and year of publication. Clarithromycin resistance increased from 29.1% (2015–2019) to 36.5% (2020–2023).

**Conclusion:**

This study highlights the critical challenges of macrolide resistance in treating *H. pylori* infection. The high prevalence and geographic variation underscore the need for tailored treatment strategies based on regional resistance patterns. Furthermore, continuously monitoring resistance trends and investigating contributing factors are essential to optimize treatment.

**Systematic review registration:**

https://www.crd.york.ac.uk/prospero; CRD42024557749.

## 1 Introduction

*Helicobacter pylori* infection is a significant global health burden, affecting nearly half the world’s population (Elbehiry et al., 2023). Chronic bacterial colonization of the gastric mucosa can lead to persistent inflammation, resulting in gastrointestinal diseases, including peptic ulcers, gastric cancer, and mucosa-associated lymphoid tissue (MALT) lymphoma (Youssefi et al., 2021). In 1994 and again in 2009, *H. pylori* was classified as a class I carcinogen by the International Agency for Research on Cancer (IARC) (Ahn and Lee, 2015). Gastric cancer is the third most common cause of cancer-related morbidity worldwide and is the fifth most prevalent malignancy, accounting for 9% of all cancer-related deaths. Eradication of *H. pylori* infection effectively reduces gastritis and the risk of associated complications, making it a cost-effective strategy for the prevention of gastric cancer (Wu et al., 2019). Current treatment regimens for *H. pylori* infection primarily include triple therapy, consisting of a proton pump inhibitor (PPI) combined with two antibiotics, or quadruple therapy, which adds a bismuth agent to the PPI and antibiotics (Aumpan et al., 2023). Metronidazole, amoxicillin, clarithromycin, and tetracycline are frequently included in triple or quadruple regiments (Kim and Chung, 2020). However, the widespread use of antibiotics in clinical practice has increased antimicrobial resistance among *H. pylori* strains, particularly macrolides such as clarithromycin. While multiple bacterial and host-related factors influence the success of *H. pylori* treatment, antimicrobial resistance remains an undeniable and critical challenge (Ali and AlHussaini, 2024). The emergence of clarithromycin-resistant strains has significantly reduced the eradication rate of *H. pylori* (Lee et al., 2005). Macrolide resistance in *H. pylori* is mainly due to modification of the target site in the 23S rRNA components of the bacterial ribosome, which prevents effective binding of macrolides by methylation or point mutations in the peptidyl transferase region of domain V of the 23S rRNA (Ayaş et al., 2024).

Moreover, clarithromycin-based regimens are now generally considered unsuitable for widespread use due to low eradication rates (<80%) (Moss et al., 2023). The standard triple therapy achieved a 90% success rate for the clarithromycin-susceptible strains, while for the clarithromycin-resistant strains, the success rate was only 22% (Luther et al., 2010). This increase in resistance significantly reduces the efficacy of *H. pylori* eradication therapy (Al-Fakhrany and Elekhnawy, 2023).

In addition, *H. pylori* has cross-resistance to macrolide antimicrobials, and prior macrolide use is often associated with the prevalence of clarithromycin-resistant strains (Gerrits et al., 2006; Xia et al., 1996). Reports from Taiwan indicate that restricting the use of macrolide antibiotics decreases the prevalence of clarithromycin-resistant strains. In many countries, resistance rates to clarithromycin have been as high as 30%. Hence, the American College of Gastroenterology recommends that if it is impossible to estimate the prevalence of the clarithromycin-resistant strains in a region, the choice of antibiotic should be based on the patient’s prior macrolide exposure (Chey et al., 2017). In particular, 14-day triple therapy with clarithromycin should be restricted to individuals in regions where clarithromycin resistance is below 15%, and there is no prior record of macrolide use (Ishibashi et al., 2023).

With dynamic changes in the epidemiology of *H. pylori* and the increasing issue of macrolide-resistant strains, a new approach is needed for effective management. Hence, the primary objective of this study was to systematically review and synthesize available data on the prevalence of macrolide resistance in *H. pylori*. To ensure the data reflected current trends, we limited the search to studies published between 2015 and 2023. This allowed us to estimate the prevalence without relying on overly outdated isolates while capturing the potential effects of the COVID-19 pandemic on resistance trends.

The secondary objectives of this study were to identify trends and changes in resistance patterns over time, explore recent developments in *H. pylori* resistance, and investigate the impact of the COVID-19 pandemic on testing, antibiotic usage, and resistance patterns. Additionally, we aimed to explore heterogeneity in resistance rates across regions and populations and assess the influence of testing methods and guidelines on the reported resistance rates. By addressing these objectives, this study aims to fill existing knowledge gaps and provide comprehensive insights into the dynamics of macrolide resistance in *H. pylori*, ultimately guiding future research and clinical practice.

## 2 Methods

Our study rigorously adhered to the Preferred Reporting Items for Systematic Reviews and Meta-Analyses (PRISMA) guidelines (Page et al., 2021) to ensure a robust meta-analytical synthesis of findings on *H. pylori* strains macrolide resistance. Our enrollment in the PROSPERO Registry (CRD42024557749) underscores this adherence and confirms our commitment to transparency and methodological integrity.

### 2.1 Eligibility criteria

The eligibility criteria for inclusion in this meta-analysis were studies that investigated *Helicobacter pylori* macrolide resistance, reported the resistance rate, provided clear sample size determination, and had full-text articles available in English. We included only cross-sectional studies that provided antimicrobial resistance (AMR) data, specifically those reporting baseline resistance results before any interventions. These studies offer a population-based snapshot of resistance rates at a specific point in time, making them ideal for estimating the prevalence of macrolide resistance. The exclusion criteria included studies published in languages other than English, review articles, case reports, case series studies, cohort studies, and pharmacokinetic studies.

### 2.2 Information sources and search strategy

To ensure a comprehensive and inclusive scope for our systematic review, we searched leading online databases, including Scopus, PubMed, Web of Science, and Embase. We selected these databases for their extensive and detailed coverage of the biomedical literature, ensuring that our review included diverse, relevant studies published between May 2015 and December 2023. The search syntax for each database was adapted according to the guidelines provided. The search included relevant keywords such as macrolide, antibiotic resistance, and *H. pylori*, as well as related MeSH terms.

### 2.3 Selection process

The results of the database searches were imported into EndNote (version 20), and duplicate entries were removed. To minimize bias, four authors (E-P, S-M-K, M-B, and N-G) independently screened the titles, abstracts, and full texts of the identified publications to assess their eligibility for inclusion in the meta-analysis. Any discrepancies in the selection process were resolved by consultation with another author (T. N), adjudicated, and the final decision was made.

### 2.4 Data collection process

Two authors (M-M and M-B) performed the data extraction independently to verify accuracy and resolve disagreements by mutual agreement.

### 2.5 Data items

The extracted data included details of the first author(s), publication year, country, diagnostic method, sample source, number of positive tests, and total number of individuals (sample size).

### 2.6 Risk of bias assessment

The quality of the included articles was assessed using the Joanna Briggs Institute (JBI) tool (Migliavaca et al., 2020). Two authors (S-M-K and M-B) independently performed the assessment process, while a third author (M-SH) investigated any discrepancies and made a decision. A score ranging from 0 to 9 points was attributed to each study (8–9 points: high quality, 6–7 points: Moderate quality, under 5 points: low quality).

### 2.7 Effect measures

This meta-analysis examined the prevalence of antibiotic resistance by examining the percentage of resistant isolates in multiple research studies. Subgroup analyses and meta-regression were used to identify the factors that cause variation in resistance rates, considering elements such as country of origin. The study also examined the time trend of macrolide resistance.

### 2.8 Synthesis methods

Proportions were used as the outcome measure. The primary objective of this study was to determine the prevalence of antibiotic-resistant bacterial strains. The secondary objective was to identify potential sources of differences between groups by performing subgroup analysis and regression focusing on countries. In addition, we examined trends in antibiotic resistance rates over time.

A random effects model was used to analyze the data. The amount of heterogeneity (τ^2^) was estimated using the DerSimonian-Laird estimator (DerSimonian and Laird, 1986). The *Q*-test for heterogeneity and the *I*^2^ statistic were also reported (Huedo-Medina et al., 2006). If heterogeneity was detected (i.e., τ^2^ > 0, regardless of the *Q*-test results), a meta-regression analysis was performed regardless of the *Q*-test results to examine the trend of antibiotic resistance rates over time. Studentized residuals and Cook’s distances were used to identify potential outliers and influential studies within the model (Zhang, 2016). Studies with a studentized residual greater than the 100 × (1–0.05/(2 × k)) percentile of a standard normal distribution (using a Bonferroni correction with two-sided *α* = 0.05 for k studies included in the meta-analysis) were considered potential outliers. Studies with a Cook’s distance greater than the median plus six times the interquartile range of Cook’s distances were considered influential. Funnel plot asymmetry was assessed using rank correlation and regression tests with the standard error of the observed outcomes as the predictor (Sterne and Egger, 2005). The analysis was performed using R (version 4.2.1) (R Core Team, 2024) and the meta for package (version 3.8.1) (R Core Team, 2018; Viechtbauer, 2010).

### 2.9 Reporting bias assessment and certainty assessment

We used rank correlation and Egger’s regression tests to assess funnel plot asymmetry and potential reporting bias. To further strengthen our results against publication bias, we implemented the Fail-Safe N and Trim-and-Fill methods, increasing the reliability and credibility of our conclusions.

## 3 Results

### 3.1 Study selection

**Figure 1:**
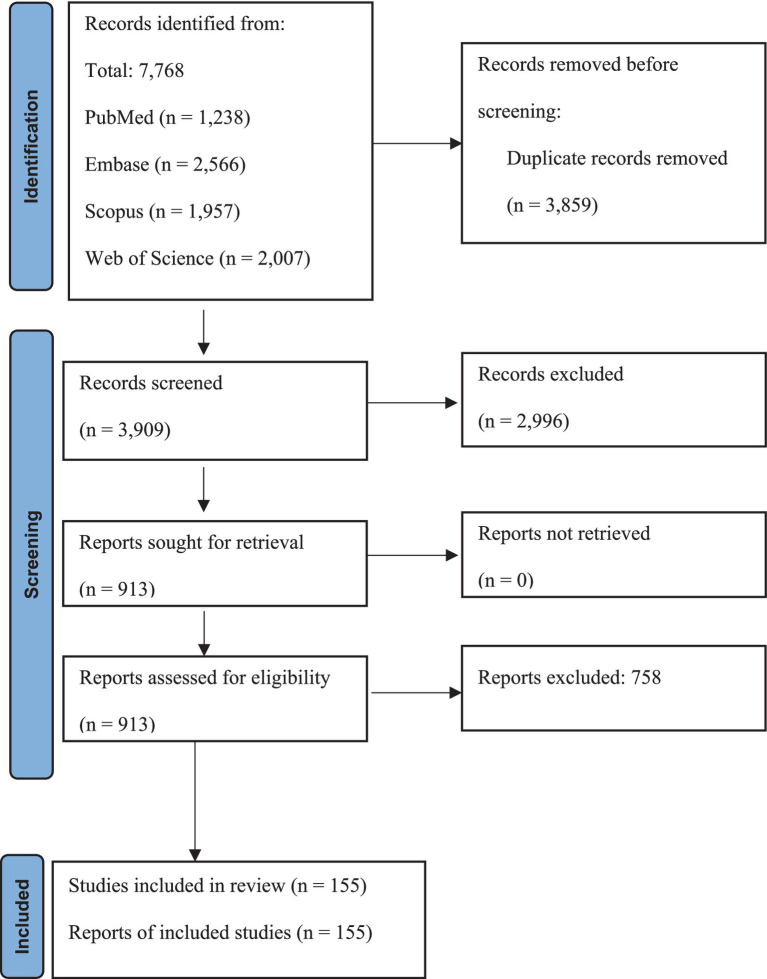
PRISMA flow chart summarizes the article selection procedure. Flow diagram illustrating the article selection process, including the identification, screening, eligibility, and inclusion of studies.

A systematic online database search yielded 7,768 records. After eliminating 3,859 duplicates, 3,909 articles were initially screened in the abstract section. Subsequently, 913 articles were evaluated in depth, and 758 were excluded based on specific criteria. Finally, this systematic review and meta-analysis included 155 eligible studies (178 reports). Characteristics and references of included studies are presented in Supplementary Table S1. The screening and selection processes are summarized in the PRISMA flowchart (Figure 1).

### 3.2 Study characteristics

Overall, the meta-analysis included a total of 155 studies conducted between the years 2015 and 2023 from various regions across the globe. Most of the reports were sourced from Asia (*n* = 94), followed by Europe (*n* = 28), America (*n* = 12), Africa (*n* = 6), and, finally, Australia (*n* = 2). Also, one study was conducted simultaneously on different continents. The studies included in this review were performed in 43 countries: China, South Korea, Japan, Slovenia, Iran, Vietnam, Romania, India, the United States, Italy, Israel, Spain, Russia, Jordan, Turkey, Canada, Indonesia, Cameroon, Singapore, Pakistan, Mexico, Bangladesh, Germany, Taiwan, Switzerland, Malaysia, Belgium, Algeria, Saudi Arabia, Poland, Tunisia, France, Venezuela, Bhutan, Cambodia, Chile, Bulgaria, Austria, Colombia, Thailand, Egypt, Australia, and Portugal. Among the guidelines used to interpret antimicrobial susceptibilities, CLSI (Clinical & Laboratory Standards Institute) and EUCAST (European Committee on Antimicrobial Susceptibility Testing) were widely used in most studies (44 and 77 reports, respectively). The JBI critical appraisal checklist was utilized to evaluate the characteristics of the reviewed studies. Out of the 155 included studies, 147 were low risk, and 8 were some risks.

### 3.3 Prevalence of erythromycin resistance

**Figure 2:**
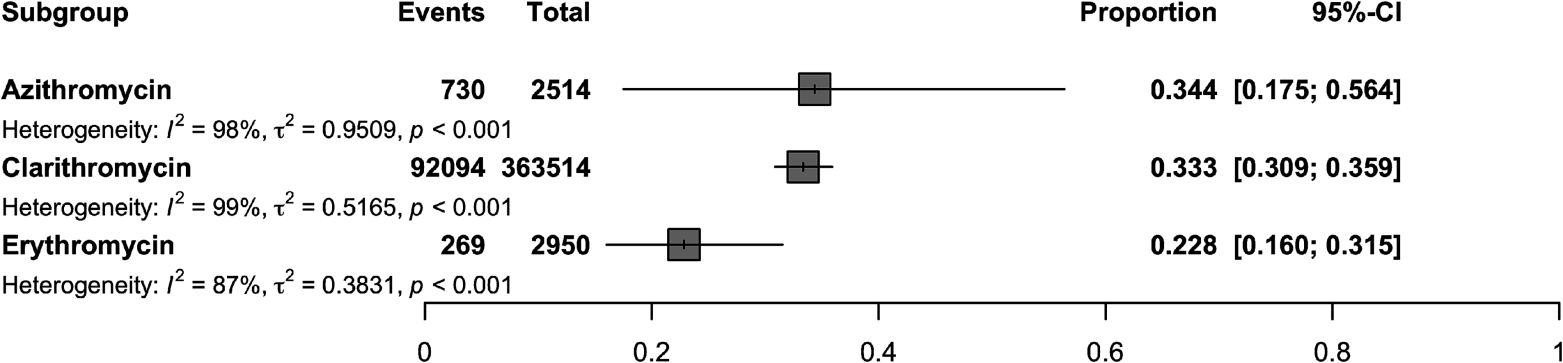
Forest plot illustrating the overall proportion of antibiotic resistant *H. pylori* isolates (Erythromycin, Clarithromycin, Azithromycin) estimated using a random-effects model.

A total of 2,950 *H. pylori* isolates from 10 studies were included in erythromycin resistance analysis. Based on the random effects model, the estimated average proportion was 0.228 (95% CI [0.160, 0.315]) (Table 1). Therefore, the average outcome differed significantly from zero (*z* = −5.395, *p* < 0.001). According to the *Q* test, accurate outcomes were heterogeneous (Q (9) = 71.005, *I*^2^ = 87.32%, *p* < 0.001). The forest plot in Figure 2 shows the observed outcomes and the model estimates. With the implementation of the fill and trim method, the proportion changed to 0.289 (95% CI [0.206, 0.389]). An examination of the studentized residuals revealed that one study (Hu et al., 2023) had a value more considerable of 2.807 and maybe a potential outlier in the context of this model. After excluding this potential outlier, the proportion was found to be 0.289 (95% CI, 0.206, 0.389). According to Cook’s distance, none of the studies could be considered overly influential. Neither the rank correlation nor the regression test indicated funnel plot asymmetry (*p* = 0.136 and *p* = 0.464, respectively) (Table 2 and Supplementary Figure S1).

**Table 1:** Meta-analysis statistics for worldwide antibiotic resistance in *H. pylori*.

Antibiotic	Egger test	Begg test	Fail and safe	Trim and fill
Erythromycin	*p* < 0.001	*p* = 0.037	757	0.289 (0.206, 0.389)
Clarithromycin	*p* < 0.001	*p* < 0.001	386,694	0.427 (0.392, 0.463)
Azithromycin	*p* = 0.446	*p* = 0.268	314	0.381 (0.210, 0.588)

This table provides a comprehensive assessment of potential publication bias in the meta-analysis using a range of statistical techniques. Included are statistics generated from Egger’s Method, Begg’s Method, the Fail-Safe N (NFS), and the Trim-and-Fill Method. These methods are applied to investigate the presence of bias and its impact on the meta-analysis results, ensuring the robustness and reliability of the findings.

**Table 2:** Evaluation of publication bias in meta-analysis.

Antibiotic	K (n, N)	Proportion 95%CI (LCI, HCI)	*I* ^2^	P1	P2
Erythromycin	10 (269, 2,950)	0.228 (0.160, 0.315)	87.32%	*p* < 0.001	*p* < 0.001
Clarithromycin	178 (92,094, 363,514)	0.333 (0.309, 0.359)	98.57%	*p* < 0.001	*p* < 0.001
Azithromycin	5 (730, 2,514)	0.344 (0.175, 0.564)	98.35%	*p* = 0.161	*p* < 0.001

K, Number of reports; n, Number of resistant isolates; N, Number of total isolates; LCI, 95% Lower Limit Confidence Interval; HCI, 95% Higher Limit Confidence Interval; P1, *p*-value of difference from zero resistance rate; P2, *p*-value of heterogeneity between reports.

### 3.4 Prevalence of clarithromycin resistance

The 363,514 *H. pylori* isolates investigated from 155 studies (by 178 reports) were included in the clarithromycin resistance analysis. Based on the random effects model, the estimated average proportion was 0.333 (95% CI [0.309, 0.359]) (Table 1). Therefore, the average outcome differed significantly from zero (*z* = −11.959, *p* < 0.001). According to the *Q*-test, the actual outcomes were heterogeneous (Q (177) = 12380.201, *I*^2^ = 98.57%, *p* < 0.001). With the implementation of the fill and trim method, the proportion changed to 0.427 (95% CI [0.392, 0.463]), and an examination of the studentized residuals revealed that several studies (Vilaichone et al., 2020) had values more significant than 3.632 and may be potential outliers in the context of this model. After excluding these potential outliers, the proportion was 0.427 (95%CI [0.392, 0.463]). Based on Cook’s distance, several studies can be considered overly influential. After removing potential outliers, the proportion was 0.427 (95% CI [0.392, 0.463]). Neither the rank correlation nor the regression test indicated funnel plot asymmetry (*p* = 0.195 and *p* = 0.586, respectively) (Table 2 and Supplementary Figure S1).

### 3.5 Prevalence of azithromycin resistance

A total of 2,514 isolates from four studies were included in analyzing azithromycin resistance. Based on the random effects model, the estimated average proportion was 0.344 (95% CI [0.175, 0.564]) (Table 1). Therefore, the average outcome was not significantly different from zero (*z* = −1.402, *p* = 0.161). The *Q*-test indicated that the actual outcomes were heterogeneous (Q (4) = 242.935, *I*^2^ = 98.35%, *p* < 0.001). After implementing the fill-and-trim method, the proportion changed to 0.381 (95% CI [0.210, 0.588]). An examination of the studentized residuals revealed that one study (Hu et al., 2023) had a value greater than 2.576 and maybe a potential outlier in the context of this model. Excluding this potential outlier, the proportion was 0.381 (95% CI [0.210, 0.588]). According to Cook’s distance method, one study (Zhang et al., 2020) can be considered overly influential. Neither the rank correlation nor the regression test indicated funnel plot asymmetry (*p* = 0.083 and *p* = 0.521, respectively) (Table 2 and Supplementary Figure S1).

### 3.6 Subgroup analysis

This section provides a comprehensive overview of the subgroup analyses of antibiotic resistance. Supplementary Table S2 presents the complete data. The analyses investigated differences in resistance rates based on geography, AST methods, trends over time, and study quality.

#### 3.6.1 Subgroup analysis based on countries

**Figure 3:**
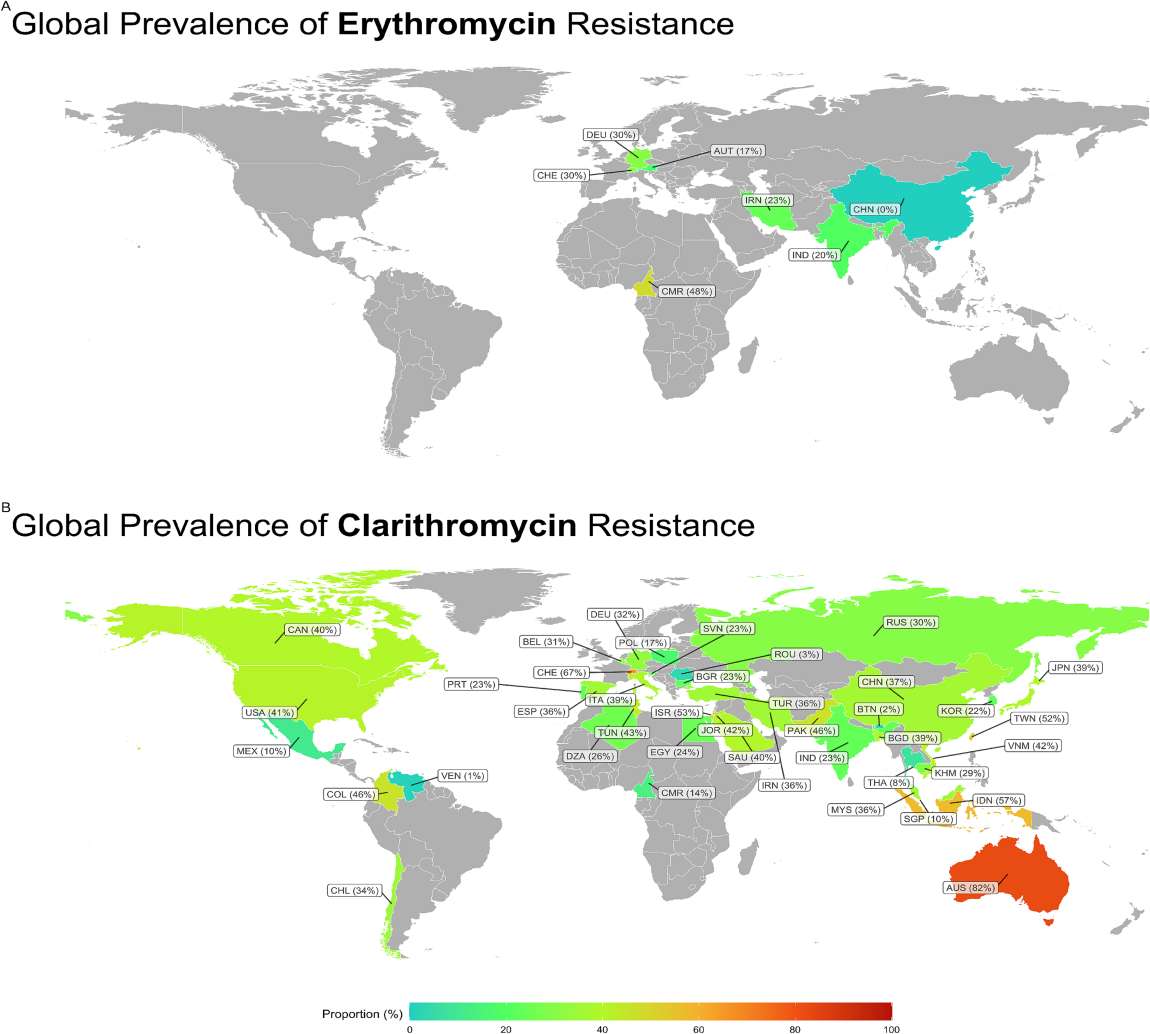
Global prevalence of Erythromycin and Clarithromycin resistance in *H. pylori*. Clarithromycin resistance was lowest in Venezuela (1%) and highest in Australia (81.9%). Erythromycin resistance was absent in China, whereas Cameroon reported the highest rate (47.9%). CAN, Canada; USA, United States; MEX, Mexico; COL, Columbia; VEN, Venezuela; CHL, Chili; DEO, Denmark; BEL, Belgium; CHE, Switzerland; PRT, Portugal; ESP, Spain; POL, Poland; ITA, Italy; SVN, Slovenia; BGR, Bulgaria; ROU, Romania; RUS, Russia; TUN, Tunisia; DZA, Algeria; EGY, Egypt; CMR, Cameroon; ISR, Israel; JOR, Jordan; SAU, Saudia; IRN, Iran; TUR, Turkey; PAK, Pakistan; IND, India; CHN, China; BTN, Kingdom of Bhutan; BGD, Bangladesh; THA, Thailand; MYS, Malaysia; SGP, Singapore; COR, South Korea; JPN, Japan; TWN, Tiwan; VNM, Vietnam; KHN, Cambodia; IDN, Indonesia; AUS, Australia.

**Figure 4:**
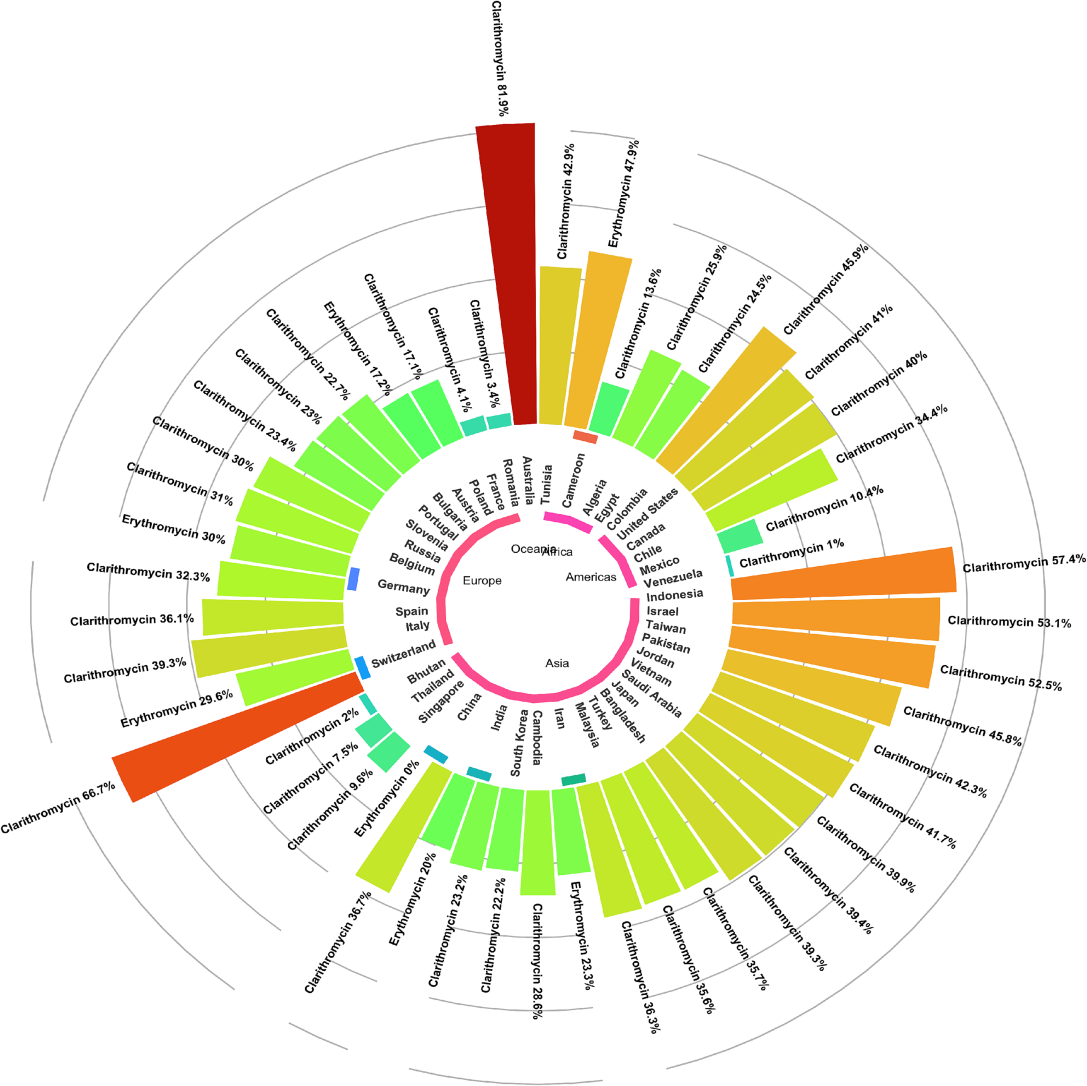
Global prevalence of macrolide-resistant *Helicobacter pylori* strains, categorized by resistance to clarithromycin and erythromycin across different regions.

Subgroup analysis revealed a significant difference in the prevalence of antibiotic resistance, particularly for clarithromycin and erythromycin, between different countries. Venezuela had the lowest clarithromycin resistance rate (1%), while Australia had the highest rate (81.9%). China had no strains resistant to erythromycin, while Cameroon had the highest rate at 47.9% (Figures 3, 4).

#### 3.6.2 Subgroup analysis based on continents

**Figure 5:**
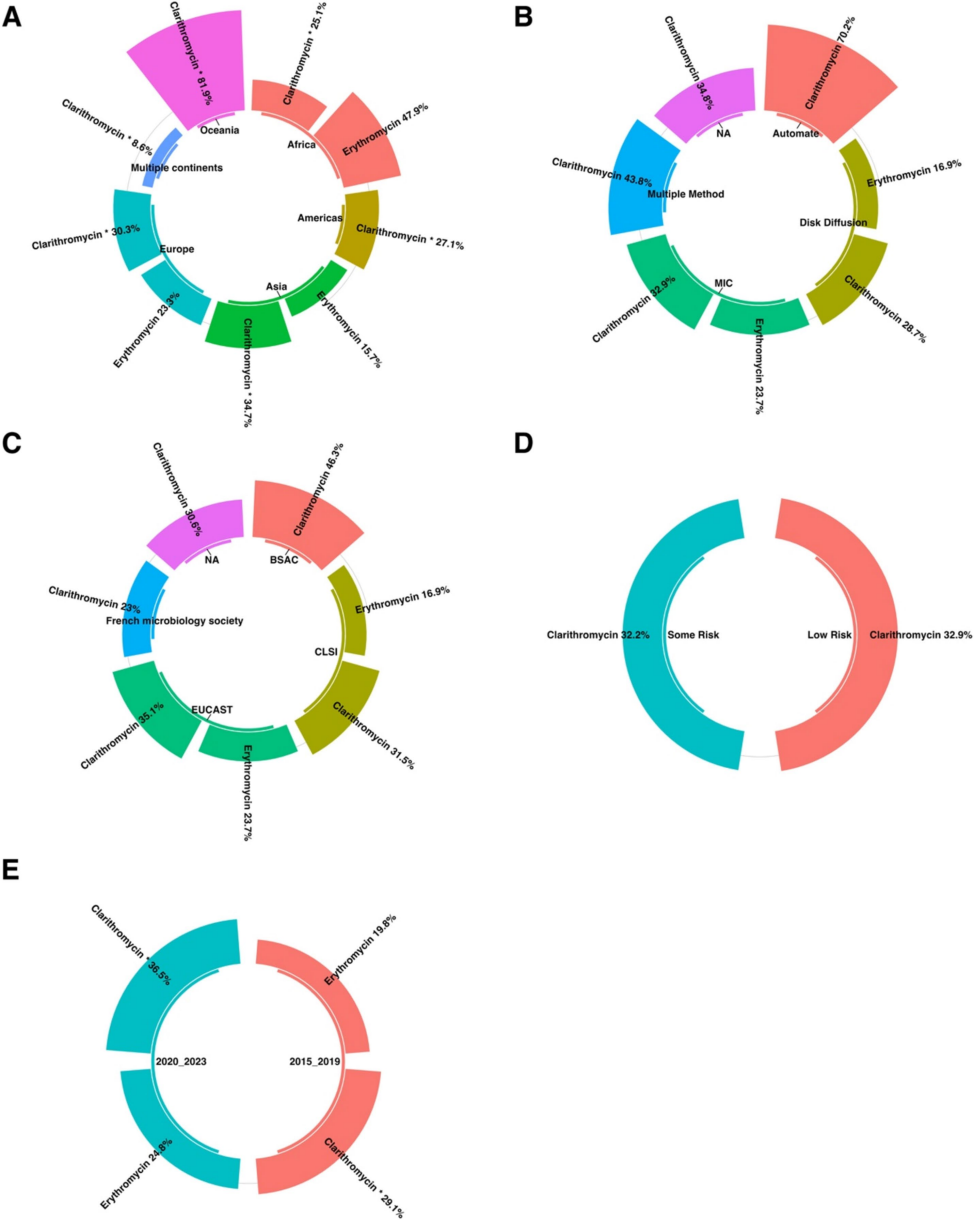
The subgroup analysis results of clarithromycin-resistant *H. pylori* isolates are presented as follows: **(A)** Comparison of prevalence by continent, with Oceania having the highest resistance rate (81.9%) and the Americas having the lowest (27.1%). **(B)** Comparison of prevalence by AST shows no statistically significant difference in resistance prevalence across the different AST methods. **(C)** A comparison of the AST guidelines revealed no significant difference in the prevalence of resistance among the various guidelines. **(D)** Comparison by risk of bias using the JBI tool indicated no statistically significant difference in resistance prevalence among the quality groups. **(E)** Comparison of erythromycin resistance before and after 2020 showed a significant difference, with the 2015–2019 cohort having the lowest clarithromycin resistance rate (29.1%) and the 2020–2023 cohort showing the highest rate (36.5%).

Subgroup analysis showed a significant difference in the prevalence of antimicrobial resistance, particularly for clarithromycin, between continents. Oceania had the highest resistance rate (81.9%), while the Americas had the lowest rate (27.1%) (Figures 4, 5A).

#### 3.6.3 Subgroup analysis based on AST method

Subgroup analysis revealed no statistically significant difference in the prevalence of antibiotic resistance between the different AST methods (Figure 5B).

#### 3.6.4 Subgroup analysis based on AST guideline

Subgroup analysis revealed no statistically significant difference in the prevalence of antibiotic resistance among the various AST guidelines (Figure 5C).

#### 3.6.5 Subgroup analysis based on quality group

Subgroup analysis revealed no statistically significant difference in the prevalence of antibiotic resistance among the various quality groups (Figure 5D).

#### 3.6.6 Subgroup analysis based on year-group

Subgroup analysis revealed a significant difference in the prevalence of antibiotic resistance, particularly to clarithromycin, across different year groups. The 2015–2019 cohort had the lowest resistance rate to clarithromycin (29.1%). Conversely, the highest resistance rate (36.5%) was observed in the 2020_2023 group (Figure 5E).

### 3.7 Meta-regression

**Figure 6:**
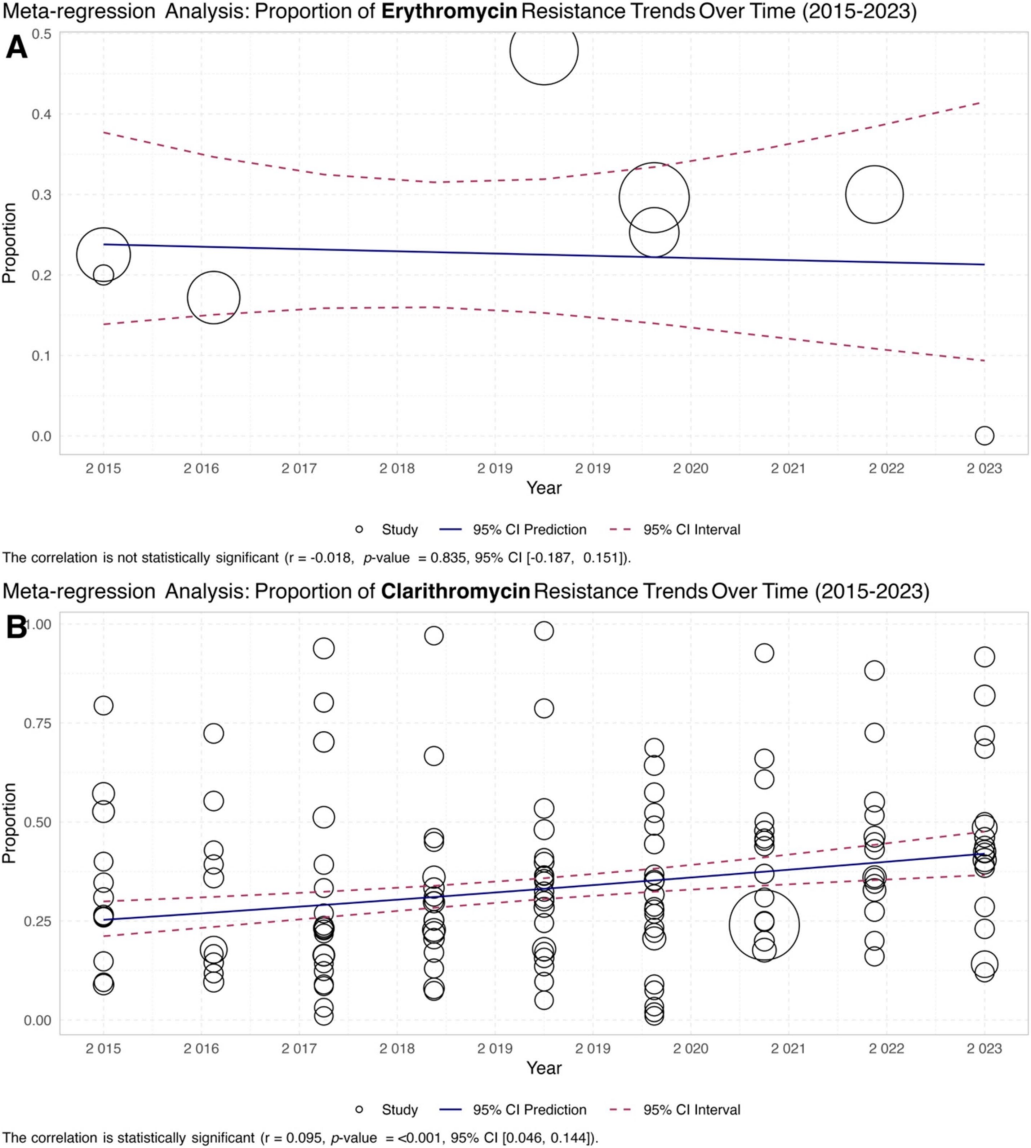
Meta-regression analysis of macrolide resistance in *H. pylori* isolates from 2015 to 2023. **(A)** Scatter plot illustrating the trend of erythromycin-resistant isolates, showing a stable resistance rate with a correlation coefficient of −0.0194 and a non-significant *p*-value of 0.885. **(B)** The scatter plot showed a significant upward trend in clarithromycin resistance, with a positive correlation coefficient of 0.056 and a p-value of less than 0.001.

The meta-regression analysis of macrolide resistance in *H. pylori* from 2015 to 2023 shows contrasting trends for erythromycin and clarithromycin. In Figure 6A, erythromycin resistance has remained relatively stable, as indicated by the correlation coefficient of −0.0194 and a non-significant *p*-value of 0.885. This suggests that there has been no significant change in the proportion of erythromycin-resistant *H. pylori* over the period analyzed. Conversely, Figure 6B shows a notable increase in clarithromycin resistance, with a positive correlation coefficient of 0.056 and a *p*-value of less than 0.001, indicating a significant upward trend. This increasing resistance to clarithromycin highlights the growing challenge of treating *H. pylori* infection and underscores the need for ongoing surveillance and adjustments in antibiotic therapy protocols (Figure 6).

## 4 Discussion

This systematic review and meta-analysis comprehensively analyze macrolide resistance prevalence and time trends in *H. pylori*, particularly on clarithromycin. By analyzing data from 155 studies in countries from 2015 to 2023, this meta-analysis provides an in-depth understanding of the global prevalence of macrolide resistance in *H. pylori*. In some countries, clarithromycin is still considered a first-choice antibiotic for treating *H. pylori* infection (Kocsmár et al., 2021). Table 3 presents a summary box that highlights the current knowledge and the new findings from this study. In this meta-analysis, the majority of studies (178 reports from 42 countries) assessed clarithromycin susceptibility, with a pooled prevalence of 33.3%. However, there was high heterogeneity between studies (*I*^2^ = 95.22% and *p* < 0.001) (Table 1). Also, the funnel plot and Egger’s test showed evidence of publication bias in some studies (p < 0.001), so the pooled prevalence changed to 42.7% after fill and trim analysis. This publication bias may be due to differences in the study population, period, sampling, methodology and guidelines used for antibiotic susceptibility testing, and the specific anatomical location of the gastric biopsy (corpus or antrum).

**Table 3:** A summary box from the current knowledge and the new findings from this study.

Knowledge from previous meta-analysis reviews	Finding the current meta-analysis review (2015–2023)
The prevalence of resistance to clarithromycin in various continents	
16.1% in Oceania (1996–2013) (Schubert et al., 2021)	81.9% in Oceania
21% in Asia (1990–2019) (Sholeh et al., 2023)	34.7% in Asia
35.97% in Asia (2011–2021) (Khoshnood et al., 2023)	27.1% in the Americas
12% in Latin America (1998 to 2011) (Camargo et al., 2014), 29.2% in African (1986–2017) (Jaka et al., 2018)	25.1% in African
18% in America (2007 to 2017) (Savoldi et al., 2018), 31.5% in America (2011 to 2021) (Ho et al., 2022)	30.3% in Europe
The prevalence of resistance to clarithromycin during time periods	
24.28% in 2010–2017 and 32.14% in 2018–2021 (Sholeh et al., 2020)	29.1% from 2015 to 2019 and 36.5% from 2020 to 2023
13% in 2006–2008 and 21% in 2012 to 2016 (Savoldi et al., 2018)	

Subgroup analyses revealed a significant variation in the prevalence of clarithromycin resistance among different geographic regions. The Oceania continent had the highest level of resistance to clarithromycin (81.9%, two reports) compared to other continents. This finding was consistent with the results of the meta-analysis conducted by Khoshnood et al. (2023), which indicated a high prevalence of clarithromycin-resistant *H. pylori* in this continent from 2011 to 2021. In contrast to the findings mentioned above, two meta-analyses conducted by Kuo et al. (2021) and Schubert et al. (2021) indicated a very low prevalence of resistance to clarithromycin in Australia between 1990–2015 and 1996–2013, respectively. The increasing trend of resistance to clarithromycin in recent years may be due to the indiscriminate use of this antibiotic and the potential for cross-resistance between macrolides.

Based on this meta-analysis, most reports (178 reports) were from Asia with a pooled prevalence of 34.4%, particularly from China, Iran, and South Korea with 39, 17, and 8 reports and a pooled prevalence of 36.7, 36.3, and 22.2%, respectively. In a previous meta-analysis in Iran, we showed a 21% prevalence of resistance to clarithromycin from 1990 to 2019 (Sholeh et al., 2020). Consistent with our results, a meta-analysis conducted by Khoshnood et al. (2023) also showed a similar prevalence of resistance to clarithromycin in Asia during the 2011–2021 period (35.97%). A meta-analysis review showed a prevalence of 23.76% (22 reports) of primary resistance to clarithromycin in *H. pylori* isolated from China from 2005 to 2022 (Wang et al., 2023). On the other hand, two independent meta-analyses conducted by Ayaş and Gürol (2023) and Sarıkaya et al. (2024) showed lower rates of clarithromycin-resistant *H. pylori* in Turkey compared to the results of our meta-analysis for this country (35.7%, seven reports). In addition, Ayaş and Gürol (2023) and Sarıkaya et al. (2024) found a pooled prevalence of 26.7% (20 reports) and 30.5% (34 reports) of clarithromycin-resistant *H. pylori* during the periods from 2005 to 2020 and from 2002 to 2021, respectively. This discrepancy in results reflects differences in the periods of the studies included, the use of different methodologies and guidelines for determining antibiotic susceptibility, and various levels of consumption of macrolides in each region for the treatment of respiratory and gastric infections.

According to the results of this meta-analysis, the American continent had lower rates of clarithromycin-resistant *H. pylori* than Asia (27.1% vs. 34.7%). However, the rates of resistance to clarithromycin in the USA (41%, five reports) and Canada (40%, one report) were higher than the average prevalence for this continent. In addition, two meta-analyses conducted in Latin America and the Americas by Camargo et al. (2014) and Savoldi et al. (2018) showed the pooled prevalence of resistance to clarithromycin of 12% (35 reports, from 1998 to 2011) and 18% (13 reports, from 2007 to 2017), respectively, which were significantly lower than our meta-analysis. However, in a meta-analysis conducted in America, Ho et al. (2022) found higher rates of clarithromycin-resistant *H. pylori* during 2011–2021 compared to the present meta-analysis (31.5% vs. 27.1%). These findings highlight an increasing trend of clarithromycin-resistant *H. pylori* in recent years in this continent.

This meta-analysis showed that the African continent had the lowest prevalence of resistance to clarithromycin (25.1%, nine reports). However, Jaka et al. (2018) in a meta-analysis study, showed a higher prevalence of clarithromycin-resistant *H. pylori* in this continent from 1986 to 2017 (29.2%, 26 reports) than our results. A systematic review with meta-analysis conducted by Hooi et al. (2017) showed that the African continent had the highest rate of *H. pylori* infection, with a prevalence of 70.1% from 1970 to 2016. Nevertheless, there needs to be more data on the antibiotic susceptibility of this bacterium in Africa due to the lack of culture facilities and antibiotic susceptibility testing in most regions. Therefore, it is necessary to set up laboratory facilities on a large scale to monitor the antimicrobial resistance of *H. pylori* in this continent. Moreover, there is an alarm for the prevalence of more than 50% of clarithromycin-resistant *H. pylori* in Switzerland, Taiwan, Indonesia, and Israel. This alarming trend highlights the urgent need for increased surveillance and development of alternative treatment strategies to combat antimicrobial resistance, ensure effective treatment of *H. pylori* infections, and minimize associated health risks worldwide.

Year subgroup analysis showed that the prevalence of resistance to clarithromycin increased significantly over two different periods (29.1% from 2015 to 2019 and 36.5% from 2020 to 2023). This may be due to the extensive use of macrolides in treating respiratory tract infections, particularly during the COVID-19 pandemic. Consistent with these findings, a previous meta-analysis in Iran demonstrated a significant increase in the prevalence of clarithromycin-resistant *H. pylori* from 24.28% in 2010–2017 to 32.14% in 2018–2021 (Sholeh et al., 2020). Also, Savoldi et al. (2018), in a meta-analysis, demonstrated an increasing trend of prevalence of clarithromycin in *H. pylori* in all continents over three different periods (2006 to 2008, 2009 to 2011, and 2012 to 2016).

Subgroup analysis of the AST (Antimicrobial Susceptibility Testing) guidelines revealed that studies using the EUCAST guideline reported higher levels of clarithromycin resistance compared to those using the CLSI guideline (35.1% vs. 31.5%). Additionally, more studies followed the EUCAST guideline than the CLSI guideline (87 vs. 48 reports). These two widely used guidelines have different breakpoints for defining resistance, with EUCAST considering strains resistant at MIC >0.25 mg/mL, while CLSI defines resistance at MIC ≥1 mg/mL. These differing thresholds, along with the unequal number of studies using each guideline, likely contribute to the observed variations in clarithromycin resistance prevalence.

Furthermore, many researchers apply clarithromycin resistance criteria to other macrolides, such as azithromycin and erythromycin, to maintain consistency within the macrolide class. This practice is particularly important because specific breakpoints for these two antibiotics are not provided in either the EUCAST or CLSI guidelines.

According to the results of this meta-analysis, fewer studies evaluate the susceptibility testing of azithromycin and erythromycin compared to clarithromycin on a global scale. This discrepancy may stem from the fact that azithromycin and erythromycin are not recommended as first-line treatments in *H. pylori* eradication regimens, leading to a focus on clarithromycin in resistance studies. According to this meta-analysis, the pooled prevalence of azithromycin resistance was relatively similar to that of clarithromycin resistance (33.3% vs. 34.4); however, only four reports had determined the antibiotic susceptibility of *H. pylori*. On the other hand, the pooled prevalence of erythromycin resistance was lower than resistance to clarithromycin and azithromycin (22.8%, 10 reports). Yousefi-Avarvand et al. (2018), in a meta-analysis conducted in Iran, showed a prevalence of 19% (3 reports) of azithromycin resistance in Iranian children. It is important to note while the exact breakpoints for azithromycin and erythromycin are not determined by the CLSI or EUCAST guidelines, many scientific articles apply the clarithromycin breakpoint as a reference point for these antibiotics due to their shared class as macrolides. This practice is common across multiple studies and allows for a more consistent interpretation of resistance patterns.

This meta-analysis had several limitations, including the lack of evaluation of secondary antibiotic resistance, the failure to evaluate macrolide-heteroresistant strains, the inability to differentiate the prevalence of antibiotic resistance in adults from that in children, and the inability to assess the efficacy of regimens containing macrolides in settings with either low or high prevalence of macrolide resistance.

## 5 Conclusion

This systematic review and meta-analysis revealed a remarkably high prevalence of macrolide resistance in *H. pylori*, particularly to clarithromycin. Significant geographical differences in resistance were identified, with rates ranging from 1 to 81.9% across various regions, highlighting an upward trend. This study underscores the urgent need for tailored treatment strategies based on local resistance patterns, and the establishment of comprehensive global surveillance programs. These initiatives are essential for improving treatment effectiveness and preventing failures, as they would monitor resistance trends and investigate contributing factors such as antibiotic usage patterns and the prevalence of specific *H. pylori* strains. Future research must prioritize understanding these dynamics to develop more effective therapies to eradicate *H. pylori* infections globally.

## Data Availability

The original contributions presented in the study are included in the article/Supplementary material, further inquiries can be directed to the corresponding authors.
