# Supplementary material for: Insights into Helicobacter pylori macrolide resistance: a comprehensive systematic review and meta-analysis
Source: Front Microbiol. 2024 Oct 30;15:1481763. doi: 10.3389/fmicb.2024.1481763 (PMC11557415; doi:10.3389/fmicb.2024.1481763)
Supplement: Supplementary file 1 [file Data_Sheet_1.pdf]

The search syntax used for the literature review in each online database.

PubMed:

("Helicobacter pylori"[tiab] OR "H. pylori"[tiab]) AND (macrolide*[tiab] OR azithromycin[tiab] OR clarithromycin[tiab] OR erythromycin[tiab] OR roxithromycin[tiab] OR telithromycin[tiab] OR spiramycin[tiab] OR fidaxomicin[tiab]) AND (resistant*[tiab] OR susceptible*[tiab])

Embase:

('helicobacter pylori':ti,ab OR 'h. pylori':ti,ab) AND (macrolide*:ti,ab OR azithromycin:ti,ab OR clarithromycin:ti,ab OR erythromycin:ti,ab OR roxithromycin:ti,ab OR telithromycin:ti,ab OR spiramycin:ti,ab OR fidaxomicin:ti,ab) AND (resistant*:ti,ab OR susceptible*:ti,ab)

Scopus:

(TITLE-ABS-KEY("Helicobacter pylori") OR TITLE-ABS-KEY("H. pylori")) AND (TITLE-ABS-KEY(macrolide*) OR TITLE-ABS-KEY(azithromycin) OR TITLE-ABS-KEY(clarithromycin) OR TITLE-ABS-KEY(erythromycin) OR TITLE-ABS-KEY(roxithromycin) OR TITLE-ABS-KEY(telithromycin) OR TITLE-ABS-KEY(spiramycin) OR TITLE-ABS-KEY(fidaxomicin)) AND (TITLE-ABS-KEY(resistant*) OR TITLE-ABS-KEY(susceptible*))

Web of Science:

TS=("Helicobacter pylori" OR "H. pylori") AND TS=(macrolide* OR azithromycin OR clarithromycin OR erythromycin OR roxithromycin OR telithromycin OR spiramycin OR fidaxomicin) AND TS=(resistant* OR susceptible*)

Supplementary Table 1

Characteristics and Extracted Data of Studies Included in the Meta-analysis

Author, Date (Reference)	Countries	AST method	AST guideline	Quality. Group	Azithromycin	Clarithromycin	Erythromycin
**(Wu, Wang et al. 2017)**	China	MIC	CLSI	Low Risk	NA	10	NA
**(Shu, Ye et al. 2022)**	China	MIC	CLSI	Low Risk	NA	538	NA
**(Bang, Lim et al. 2020)**	South Korea	MIC	CLSI	Low Risk	NA	57	NA
**(Guo, Liu et al. 2019)**	China	MIC	CLSI	Low Risk	NA	8	NA
**(Seo, Park et al. 2019)**	South Korea	MIC	CLSI	Low Risk	NA	1	NA
**(Kobatake, Ogino et al. 2021)**	Japan	MIC	CLSI	Low Risk	NA	6	NA
**(Butenko, Jeverica et al. 2017)**	Slovenia	MIC	EUCAST	Low Risk	NA	25	NA

**(Hamidi, Badmasti et al. 2020)**	Iran	MIC	EUCAST	Low Risk	NA	11	NA
**(Korona-Glowniak, Cichoz-Lach et al. 2019)**	NA	MIC	EUCAST	Low Risk	NA	30	NA
**(Nguyen, Le et al. 2023)**	Vietnam	MIC	CLSI	Low Risk	NA	61	NA
**(IM 2020)**	Romania	MIC	EUCAST	Low Risk	NA	2	NA
**(Wang, Li et al. 2023)**	China	MIC	EUCAST	Low Risk	NA	117	NA
**(Wani, Bashir et al. 2018)**	India	NA	NA	Low Risk	NA	27	NA
**(Li, Ke et al. 2017)**	China	MIC	NA	Low Risk	NA	286	NA
**(Vu, Tran et al. 2022)**	Vietnam	MIC	EUCAST	Low Risk	NA	20	NA
**(Li, Deng et al. 2021)**	China	MIC	EUCAST	Low Risk	NA	24	NA
**(Shao, Lu et al. 2018)**	China	MIC	CLSI	Low Risk	NA	519	NA
**(Shiota, Reddy et al. 2015)**	United States	MIC	EUCAST	Low Risk	NA	20	NA
**(Li, Zhou et al. 2021)**	China	MIC	CLSI	Low Risk	NA	63	NA
**(Jiang, Qian et al. 2022)**	China	MIC	CLSI	Low Risk	NA	759	NA
**(Rasi-Bonab, Jafari-Sales et al. 2021)**	Iran	Disk Diffusion	CLSI	Low Risk	NA	25	NA
**(Sadeghi, Narimani et al. 2022)**	Iran	Disk Diffusion	CLSI	Low Risk	NA	10	NA
**(Fiorini, Zullo et al. 2018)**	Italy	MIC	EUCAST	Low Risk	NA	114	NA
**(Tang, Wang et al. 2022)**	China	MIC	EUCAST	Low Risk	NA	43	NA
**(Azrad, Vazana et al. 2022)**	Israel	MIC	BSAC	Low Risk	NA	250	NA
**(Bujanda, Nyssen et al. 2021)**	NA	MIC	EUCAST	Low Risk	NA	701	NA
**(Huang, Wu et al. 2023)**	China	Multiple Method	EUCAST	Low Risk	NA	90	NA
**(Mormeneo Bayo, Bellés Bellés et al. 2023)**	Spain	MIC	EUCAST	Low Risk	NA	17	NA
**(Akhtereeva, Morozova et al. 2018)**	Russia	Disk Diffusion	NA	Low Risk	NA	9	NA
**(Gunnarsdottir, Gudjonsson et al. 2017)**	NA	MIC	EUCAST	Low Risk	NA	9	NA
**(Mahmoudi, Mamishi et al. 2017)**	Iran	Disk Diffusion	CLSI	Low Risk	NA	7	NA
**(Shu, Yin et al. 2018)**	China	MIC	NA	Low Risk	NA	112	NA
**(Xu, Yun et al. 2022)**	China	MIC	CLSI	Low Risk	NA	109	NA
**(Mosites, Bruden et al. 2018)**	NA	MIC	CLSI	Low Risk	NA	238	NA
**(Burayzat, Al-Tamimi et al. 2023)**	Jordan	MIC	EUCAST	Low Risk	NA	30	NA
**(Caliskan, Tokman et al. 2015)**	Turkey	MIC	EUCAST	Low Risk	NA	34	NA

**(Liu, Wang et al. 2022)**	China	MIC	CLSI	Low Risk	NA	18	NA
**(Quek, Pham et al. 2016)**	Vietnam	MIC	EUCAST	Low Risk	NA	28	NA
**(Alarcón, Urruzuno et al. 2017)**	NA	MIC	EUCAST	Low Risk	NA	422	NA
**(Eng, Ybazeta et al. 2015)**	Canada	Disk Diffusion	EUCAST	Low Risk	NA	8	NA
**(Gehlot, Mahant et al. 2016)**	India	MIC	EUCAST	Low Risk	NA	8	NA
**(Cosme, Montes et al. 2017)**	NA	MIC	NA	Low Risk	NA	169	NA
**(Boltin, Ben-Zvi et al. 2015)**	Israel	MIC	EUCAST	Low Risk	NA	27	NA
**(Amin, Shayesteh et al. 2019)**	Iran	MIC	NA	Low Risk	NA	70	NA
**(Ji, Han et al. 2016)**	China	MIC	CLSI	Low Risk	NA	1720	NA
**(Fauzia, Miftahussurur et al. 2020)**	Indonesia	MIC	EUCAST	Low Risk	NA	58	NA
**(Long, Chen et al. 2018)**	China	MIC	NA	Low Risk	NA	16	NA
**(Zhang, Chen et al. 2015)**	China	MIC	NA	Low Risk	NA	53	NA
**(Kouitcheu Mabeku, Eyoum Bille et al. 2019)**	Cameroon	Disk Diffusion	CLSI	Low Risk	NA	19	67
**(Lee, Park et al. 2019)**	NA	MIC	EUCAST	Low Risk	NA	43	NA
**(Ang, Fock et al. 2016)**	Singapore	MIC	CLSI	Low Risk	NA	68	NA
**(Hu, Zhou et al. 2023)**	China	Disk Diffusion	CLSI	Low Risk	NA	790	0
**(Farzi, Behzad et al. 2019)**	NA	MIC	NA	Low Risk	NA	14	NA
**(Anis, Farooqi and Niaz 2021)**	Pakistan	MIC	CLSI	Low Risk	NA	22	NA
**(Abdollahi, Hashemzadeh et al. 2019)**	Iran	Disk Diffusion	CLSI	Low Risk	NA	23	NA
**(Alarcón-Millán, Fernández-Tilapa et al. 2016)**	Mexico	Disk Diffusion	CLSI	Low Risk	NA	8	NA
**(Tamayo, Montes et al. 2017)**	Spain	MIC	NA	Low Risk	NA	1986	NA
**(Miftahussurur, Cruz et al. 2017)**	NA	MIC	EUCAST	Low Risk	NA	2	NA
**(Khani, Talebi Bezmin Abadi and Mohabati Mobarez 2019)**	Iran	MIC	NA	Low Risk	NA	48	NA
**(Morimoto, Takeuchi et al. 2015)**	Japan	MIC	NA	Low Risk	NA	35	NA
**(Vagarali, Metgud et al. 2015)**	India	Disk Diffusion	CLSI	Low Risk	NA	NA	1
**(Bayati, Alebouyeh et al. 2020)**	Iran	MIC	CLSI	Low Risk	NA	27	NA
**(Chen, Huang et al. 2022)**	China	MIC	EUCAST	Low Risk	NA	248	NA
**(Aftab, Miftahussurur et al. 2016)**	Bangladesh	MIC	EUCAST	Low Risk	NA	22	NA
**(Cosme, Torrente Iranzo et al. 2019)**	Spain	MIC	NA	Some Risk	NA	582	NA

**(Wang, Zhao et al. 2018)**	China	MIC	EUCAST	Low Risk	NA	13	NA
**(Gatta, Scarpignato et al. 2018)**	Italy	MIC	EUCAST	Some Risk	NA	478	NA
**(Di Giulio, Di Campli et al. 2016)**	Italy	NA	NA	Low Risk	NA	131	NA
**(Li, Li and Tan 2021)**	China	NA	NA	Low Risk	NA	13	NA
**(Kageyama, Sato et al. 2019)**	Japan	MIC	CLSI	Low Risk	NA	100	NA
**(Helmbold, Ghebremedhin et al. 2022)**	Germany	MIC	EUCAST	Low Risk	NA	22	NA
**(Lu, Lai et al. 2019)**	Taiwan	MIC	NA	Low Risk	NA	5	NA
**(Nahm, Kim et al. 2019)**	Taiwan	MIC	NA	Low Risk	NA	11	NA
**(Regnath, Raecke et al. 2017)**	Germany	NA	NA	Low Risk	NA	141	NA
**(Goni, Tammer et al. 2022)**	Germany	MIC	EUCAST	Low Risk	NA	NA	33
**(Wang, Lv et al. 2017)**	China	MIC	NA	Low Risk	NA	10	NA
**(Alavifard, Mirzaei et al. 2021)**	Iran	MIC	EUCAST	Low Risk	NA	36	NA
**(Kim, Lee et al. 2020)**	Switzerland	MIC	EUCAST	Low Risk	NA	NA	53
**(Taghvaei, Kamali et al. 2021)**	Iran	MIC	CLSI	Low Risk	NA	43	NA
**(Song, Zhou et al. 2016)**	NA	MIC	EUCAST	Low Risk	NA	73	NA
**(Hu, Zeng et al. 2023)**	China	MIC	EUCAST	Low Risk	NA	40	NA
**(Hallur, Panigrahi et al. 2022)**	India	Multiple Method	EUCAST	Low Risk	NA	11	NA
**(Gehlot, Mahant et al. 2016)**	India	MIC	NA	Low Risk	NA	8	NA
**(Le Thi, Werkstetter et al. 2023)**	NA	Multiple Method	CLSI	Low Risk	NA	651	NA
**(Rezaei, Abadi and Mobarez 2020)**	Iran	Disk Diffusion	EUCAST	Low Risk	NA	17	NA
**(Vazirzadeh, Falahi et al. 2020)**	Iran	MIC	EUCAST	Low Risk	NA	NA	21
**(Hanafiah, Binmaeil et al. 2019)**	Malaysia	MIC	CLSI	Some Risk	NA	21	NA
**(Örsten, Yılmaz and Akyön 2023)**	Turkey	MIC	EUCAST	Low Risk	NA	42	NA
**(Van den Poel, Gils et al. 2021)**	Belgium	MIC	EUCAST	Low Risk	NA	13	NA
**(Bachir, Allem et al. 2018)**	Algeria	MIC	EUCAST	Low Risk	NA	68	NA
**(Eed, Hawash et al. 2019)**	Saudi Arabia	NA	NA	Some Risk	NA	57	NA
**(Hays, Delerue et al. 2019)**	NA	MIC	NA	Low Risk	NA	19	NA
**(Bińkowska, Biernat et al. 2018)**	Poland	MIC	NA	Low Risk	NA	29	NA
**(Kuo, Lee et al. 2021)**	Taiwan	MIC	EUCAST	Some Risk	NA	38	NA
**(Sukri, Hanafiah et al. 2022)**	NA	MIC	CLSI	Low Risk	NA	37	NA
**(Mansour, Fendri et al. 2016)**	Tunisia	MIC	EUCAST	Low Risk	NA	9	NA

**(Mansour, Fendri et al. 2016)**	France	MIC	EUCAST	Low Risk	NA	3	NA
**(Peña, Rojas et al. 2017)**	Venezuela	MIC	CLSI	Low Risk	NA	1	NA
**(Zhang, Wen et al. 2020)**	China	MIC	CLSI	Low Risk	NA	29	NA
**(Zhang, Wang et al. 2020)**	China	MIC	EUCAST	Low Risk	202	84	NA
**(Fauzia, Aftab et al. 2023)**	Bangladesh	MIC	EUCAST	Some Risk	NA	22	NA
**(Lee, Ahn et al. 2019)**	South Korea	MIC	NA	Low Risk	NA	32	NA
**(Zhang, Meng et al. 2021)**	China	NA	NA	Some Risk	NA	68089	NA
**(Redondo, Keller et al. 2018)**	Switzerland	MIC	EUCAST	Low Risk	NA	40	NA
**(Zerbetto De Palma, Mendiondo et al. 2017)**	NA	NA	CLSI	Low Risk	NA	14	NA
**(Salehi, Attaran et al. 2020)**	Iran	MIC	EUCAST	Low Risk	NA	11	NA
**(Bahmaninejad, Ghafourian et al. 2021)**	Iran	Disk Diffusion	EUCAST	Low Risk	NA	33	NA
**(Xiong, Mohammed Aljaberi et al. 2023)**	China	Disk Diffusion	EUCAST	Low Risk	NA	1606	NA
**(Lok, Zhu et al. 2020)**	China	Multiple Method	EUCAST	Low Risk	NA	34	NA
**(Camorlinga-Ponce, Gómez-Delgado et al. 2021)**	Mexico	MIC	EUCAST	Low Risk	NA	15	NA
**(Binyamin, Pastukh et al. 2017)**	Mexico	MIC	EUCAST	Some Risk	NA	15	NA
**(Chen, Cunningham et al. 2017)**	United States	Automate	CLSI	Low Risk	NA	290	NA
**(Ziver-Sarp, Yuksel-Mayda et al. 2021)**	Turkey	MIC	EUCAST	Low Risk	NA	24	NA
**(Vilaichone, Aumpan et al. 2020)**	Bhutan	MIC	EUCAST	Low Risk	NA	7	NA
**(Aumpan, Vilaichone et al. 2020)**	Cambodia	MIC	NA	Low Risk	NA	4	NA
**(Gonzalez-Hormazabal, Musleh et al. 2018)**	Chile	NA	NA	Some Risk	NA	29	NA
**(Deng, Liu et al. 2023)**	China	MIC	CLSI	Low Risk	NA	89	NA
**(Oporto, Pavez et al. 2019)**	Chile	MIC	EUCAST	Low Risk	NA	18	NA
**(Shokrzadeh, Alebouyeh et al. 2015)**	Iran	Disk Diffusion	CLSI	Low Risk	NA	29	25
**(Raaf, Amhis et al. 2017)**	Algeria	MIC	EUCAST	Low Risk	NA	9	NA
**(Bachir, Allem et al. 2018)**	Algeria	Multiple Method	EUCAST	Low Risk	NA	38	NA
**(Tang, Chen et al. 2020)**	NA	MIC	EUCAST	Low Risk	NA	52	NA
**(Liu, Wang et al. 2018)**	China	Disk Diffusion	EUCAST	Low Risk	260	247	NA
**(Zhang, Zhou et al. 2015)**	China	MIC	EUCAST	Low Risk	NA	500	NA

**(Maleknejad, Mojtahedi et al. 2015)**	Iran	Disk Diffusion	NA	Low Risk	4	2	NA
**(Manfredi, Gismondi et al. 2015)**	Italy	MIC	NA	Low Risk	NA	12	NA
**(Boyanova, Gergova et al. 2017)**	Bulgaria	MIC	EUCAST	Low Risk	NA	53	NA
**(Zollner-Schwetz, Leitner et al. 2016)**	Austria	MIC	EUCAST	Low Risk	NA	NA	22
**(Matta, Zambrano and Pazos 2018)**	Colombia	Disk Diffusion	NA	Low Risk	NA	34	NA
**(Fiorini, Zullo et al. 2018)**	Italy	MIC	EUCAST	Low Risk	NA	126	NA
**(Shoosanglertwijit, Kamrat et al. 2020)**	Thailand	MIC	EUCAST	Low Risk	NA	3	NA
**(Buran, Sürücüoğlu et al. 2022)**	Turkey	NA	NA	Low Risk	NA	50	NA
**(Hanafy and Seleem 2019)**	Egypt	Disk Diffusion	CLSI	Low Risk	NA	12	NA
**(Schubert, Ingram et al. 2023)**	Australia	MIC	EUCAST	Low Risk	NA	649	NA
**(Bruce, Bruden et al. 2019)**	United States	MIC	NA	Low Risk	NA	74	NA
**(Fiorini, Saracino et al. 2017)**	Italy	MIC	EUCAST	Low Risk	NA	93	NA
**(Choi, Jeong et al. 2019)**	South Korea	MIC	CLSI	Low Risk	NA	3	NA
**(Lee, Kim et al. 2019)**	South Korea	MIC	EUCAST	Low Risk	NA	24	NA
**(Khoury, Geffen et al. 2017)**	Israel	MIC	EUCAST	Low Risk	NA	42	NA
**(Mascellino, Oliva et al. 2020)**	Italy	MIC	EUCAST	Low Risk	NA	28	NA
**(Schwarzer, Bontems et al. 2016)**	NA	MIC	CLSI	Low Risk	NA	75	NA
**(Tong, Lv et al. 2015)**	NA	MIC	CLSI	Low Risk	NA	99	NA
**(Zhang, Zhong et al. 2018)**	NA	MIC	CLSI	Low Risk	NA	10	NA
**(Erkut, Uzun et al. 2020)**	Turkey	MIC	CLSI	Low Risk	NA	29	NA
**(Dai, Zhao et al. 2022)**	China	MIC	NA	Low Risk	NA	33	NA
**(de Arbulo, Tamayo et al. 2023)**	Spain	MIC	EUCAST	Low Risk	NA	1559	NA
**(de Arbulo, Tamayo et al. 2023)**	Spain	MIC	EUCAST	Low Risk	NA	571	NA
**(Zhou, Zhong et al. 2022)**	China	MIC	EUCAST	Some Risk	NA	31	NA
**(Bai, Zhou et al. 2015)**	China	MIC	EUCAST	Low Risk	NA	56	NA
**(Yu, Luo et al. 2019)**	China	MIC	EUCAST	Low Risk	NA	169	NA
**(Chen, Cunningham et al. 2017)**	United States	MIC	CLSI	Low Risk	NA	290	NA
**(Ziver-Sarp, Yuksel-Mayda et al. 2021)**	Turkey	MIC	EUCAST	Low Risk	NA	24	NA
**(Vilaichone, Aumpan et al. 2020)**	Bhutan	MIC	EUCAST	Low Risk	NA	7	NA
**(Aumpan, Vilaichone et al. 2020)**	Cambodia	MIC	NA	Low Risk	NA	4	NA
**(Gonzalez-Hormazabal, Musleh et al. 2018)**	Chile	NA	NA	Some Risk	NA	29	NA

**(Deng, Liu et al. 2023)**	China	MIC	CLSI	Low Risk	NA	89	NA
**(Oporto, Pavez et al. 2019)**	Chile	MIC	EUCAST	Low Risk	NA	18	NA
**(Shokrzadeh, Alebouyeh et al. 2015)**	Iran	Disk Diffusion	CLSI	Low Risk	NA	29	25
**(Raaf, Amhis et al. 2017)**	Algeria	MIC	EUCAST	Low Risk	NA	9	NA
**(Bachir, Allem et al. 2018)**	Algeria	Multiple Method	EUCAST	Low Risk	NA	38	NA
**(Tang, Chen et al. 2020)**	NA	MIC	EUCAST	Low Risk	NA	52	NA
**(Liu, Wang et al. 2018)**	China	Disk Diffusion	EUCAST	Low Risk	260	247	NA
**(Zhang, Zhou et al. 2015)**	China	MIC	EUCAST	Low Risk	NA	500	NA
**(Maleknejad, Mojtahedi et al. 2015)**	Iran	Disk Diffusion	NA	Low Risk	4	2	NA
**(Manfredi, Gismondi et al. 2015)**	Italy	MIC	NA	Low Risk	NA	12	NA
**(Boyanova, Gergova et al. 2017)**	Bulgaria	MIC	EUCAST	Low Risk	NA	53	NA
**(Zollner-Schwetz, Leitner et al. 2016)**	Austria	MIC	EUCAST	Low Risk	NA	NA	22
**(Matta, Zambrano and Pazos 2018)**	Colombia	Disk Diffusion	NA	Low Risk	NA	34	NA
**(Fiorini, Zullo et al. 2018)**	Italy	MIC	EUCAST	Low Risk	NA	126	NA
**(Shoosanglertwijit, Kamrat et al. 2020)**	Thailand	MIC	EUCAST	Low Risk	NA	3	NA
**(Buran, Sürücüoğlu et al. 2022)**	Turkey	NA	NA	Low Risk	NA	50	NA
**(Hanafy and Seleem 2019)**	Egypt	Disk Diffusion	CLSI	Low Risk	NA	12	NA
**(Schubert, Ingram et al. 2023)**	Australia	MIC	EUCAST	Low Risk	NA	649	NA
**(Schubert, Ingram et al. 2023)**	United States	MIC	NA	Low Risk	NA	74	NA
**(Fiorini, Saracino et al. 2017)**	Italy	MIC	EUCAST	Low Risk	NA	93	NA
**(Choi, Jeong et al. 2019)**	South Korea	MIC	CLSI	Low Risk	NA	3	NA
**(Lee, Kim et al. 2019)**	South Korea	MIC	EUCAST	Low Risk	NA	24	NA
**(Pichon, Tran et al. 2020)**	France	MIC	NA	Low Risk	NA	2	NA
**(Choi, Lee et al. 2021)**	South Korea	MIC	CLSI	Low Risk	NA	30	NA
**(Antunes, Oleastro et al. 2023)**	Portugal	Multiple Method	French microbiology society	Low Risk	NA	32	NA
**(Liang, Tai et al. 2020)**	Taiwan	MIC	EUCAST	Low Risk	NA	126	NA
**(Saracino, Fiorini et al. 2020)**	Italy	MIC	EUCAST	Low Risk	NA	553	NA
**(Boltin, Ben-Zvi et al. 2015)**	Israel	MIC	EUCAST	Low Risk	NA	596	NA
**(Liu, Ji et al. 2021)**	China	MIC	CLSI	Low Risk	NA	31	NA

Supplementary table 1

Supplementary Table 2: Prevalence of Antibiotic Resistance

Category	Subgroup	K (n, N)	Proportion 95%CI(LCI, HCI)	I²	P1	P2	P3
**Erythromycin**							
**Overall**	NA	10 (269, 2950)	0.228 (0.160, 0.315)	81.94%	p<0.001	p<0.001	NA
**countries**	Cameroon	1 (67, 140)	0.479 (0.397, 0.561)	0.00%	p=0.612	p>0.999	p<0.001
	China	1 (0, 1955)	0.000 (0.000, 0.004)	0.00%	p<0.001	p>0.999	
	India	1 (1, 5)	0.200 (0.027, 0.691)	0.00%	p=0.215	p>0.999	
	Germany	1 (33, 110)	0.300 (0.222, 0.392)	0.00%	p<0.001	p>0.999	
	Switzerland	1 (53, 179)	0.296 (0.234, 0.367)	0.00%	p<0.001	p>0.999	
	Iran	3 (71, 305)	0.233 (0.189, 0.284)	0.00%	p<0.001	p=0.878	
	Austria	2 (44, 256)	0.172 (0.130, 0.223)	0.00%	p<0.001	p>0.999	
**continents**	Africa	1 (67, 140)	0.479 (0.397, 0.561)	0.00%	p=0.612	p>0.999	p=0.081
	Asia	5 (72, 2265)	0.157 (0.077, 0.296)	84.07%	p<0.001	p<0.001	
	Europe	4 (130, 545)	0.233 (0.169, 0.311)	74.09%	p<0.001	p=0.009	
**AST method**	Disk Diffusion	5 (118, 2322)	0.169 (0.068, 0.363)	92.47%	p=0.002	p<0.001	p=0.819
	MIC	5 (151, 628)	0.237 (0.184, 0.300)	65.53%	p<0.001	p=0.021	
**AST Guideline**	CLSI	5 (118, 2322)	0.169 (0.068, 0.363)	92.47%	p=0.002	p<0.001	p=0.819
	EUCAST	5 (151, 628)	0.237 (0.184, 0.300)	65.53%	p<0.001	p=0.021	
**year group**	2020_2023	5 (174, 2467)	0.248 (0.140, 0.402)	91.23%	p=0.002	p<0.001	p=0.280
	2015_2019	5 (95, 483)	0.198 (0.165, 0.236)	0.00%	p<0.001	p=0.712	
**Clarithromycin**							
**Overall**	NA	89 (92094, 363514)	0.333 (0.309, 0.359)	92.55%	p<0.001	p<0.001	NA
**countries**	China	39 (77433, 314698)	0.367 (0.325, 0.411)	98.87%	p<0.001	p<0.001	p<0.001
	South Korea	8 (174, 735)	0.222 (0.161, 0.299)	74.94%	p<0.001	p<0.001	
	Japan	3 (141, 355)	0.394 (0.230, 0.587)	88.12%	p=0.281	p<0.001	
	Slovenia	1 (25, 107)	0.234 (0.163, 0.323)	0.00%	p<0.001	p>0.999	
	Iran	17 (423, 1117)	0.363 (0.280, 0.455)	87.34%	p=0.004	p<0.001	

	Vietnam	3 (109, 322)	0.417 (0.119, 0.791)	97.25%	p=0.693	p<0.001
	Romania	1 (2, 59)	0.034 (0.008, 0.126)	0.00%	p<0.001	p>0.999
	India	4 (54, 228)	0.232 (0.103, 0.442)	87.97%	p=0.015	p<0.001
	United States	5 (748, 1481)	0.410 (0.211, 0.644)	98.51%	p=0.457	p<0.001
	Italy	11 (1766, 6773)	0.393 (0.273, 0.527)	98.56%	p=0.118	p<0.001
	Israel	4 (915, 1723)	0.531 (0.429, 0.630)	91.01%	p=0.556	p<0.001
	Spain	5 (4715, 17908)	0.361 (0.140, 0.662)	99.85%	p=0.367	p<0.001
	Russia	1 (9, 30)	0.300 (0.164, 0.483)	0.00%	p=0.033	p>0.999
	Jordan	1 (30, 71)	0.423 (0.314, 0.540)	0.00%	p=0.194	p>0.999
	Turkey	7 (253, 711)	0.357 (0.309, 0.409)	48.19%	p<0.001	p=0.072
	Canada	1 (8, 20)	0.400 (0.214, 0.620)	0.00%	p=0.374	p>0.999
	Indonesia	1 (58, 101)	0.574 (0.476, 0.667)	0.00%	p=0.137	p>0.999
	Cameroon	1 (19, 140)	0.136 (0.088, 0.203)	0.00%	p<0.001	p>0.999
	Singapore	1 (68, 708)	0.096 (0.076, 0.120)	0.00%	p<0.001	p>0.999
	Pakistan	1 (22, 48)	0.458 (0.324, 0.599)	0.00%	p=0.564	p>0.999
	Mexico	3 (38, 382)	0.104 (0.072, 0.146)	25.20%	p<0.001	p=0.263
	Bangladesh	2 (44, 112)	0.393 (0.307, 0.486)	0.00%	p=0.024	p>0.999
	Germany	2 (163, 657)	0.323 (0.153, 0.558)	90.69%	p=0.136	p=0.001
	Taiwan	4 (180, 307)	0.525 (0.222, 0.811)	93.45%	p=0.883	p<0.001
	Malaysia	1 (21, 59)	0.356 (0.245, 0.485)	0.00%	p=0.029	p>0.999
	Belgium	1 (13, 42)	0.310 (0.189, 0.463)	0.00%	p=0.016	p>0.999
	Algeria	5 (162, 626)	0.259 (0.226, 0.295)	0.00%	p<0.001	p=0.792
	Saudi Arabia	1 (57, 143)	0.399 (0.322, 0.481)	0.00%	p=0.016	p>0.999
	Poland	1 (29, 170)	0.171 (0.121, 0.235)	0.00%	p<0.001	p>0.999
	Tunisia	1 (9, 21)	0.429 (0.240, 0.640)	0.00%	p=0.514	p>0.999
	France	2 (5, 210)	0.041 (0.003, 0.389)	88.14%	p=0.022	p=0.004
	Venezuela	1 (1, 96)	0.010 (0.001, 0.070)	0.00%	p<0.001	p>0.999
	Switzerland	1 (40, 60)	0.667 (0.539, 0.774)	0.00%	p=0.011	p>0.999
	Bhutan	2 (14, 714)	0.020 (0.012, 0.033)	0.00%	p<0.001	p>0.999
	Cambodia	2 (8, 28)	0.286 (0.150, 0.476)	0.00%	p=0.028	p>0.999
	Chile	4 (94, 274)	0.344 (0.290, 0.403)	0.00%	p<0.001	p=0.477

	Bulgaria	2 (106, 466)	0.227 (0.192, 0.268)	0.00%	p<0.001	p>0.999	
	Colombia	2 (68, 148)	0.459 (0.381, 0.540)	0.00%	p=0.324	p>0.999	
	Thailand	2 (6, 80)	0.075 (0.034, 0.157)	0.00%	p<0.001	p>0.999	
	Egypt	2 (24, 98)	0.245 (0.170, 0.339)	0.00%	p<0.001	p>0.999	
	Australia	2 (1298, 1584)	0.819 (0.800, 0.838)	0.00%	p<0.001	p>0.999	
	Portugal	1 (32, 139)	0.230 (0.168, 0.307)	0.00%	p<0.001	p>0.999	
**continents**	Asia	104 (80047, 322311)	0.347 (0.318, 0.377)	97.95%	p<0.001	p<0.001	p<0.001
	Europe	30 (6935, 26721)	0.303 (0.227, 0.393)	99.18%	p<0.001	p<0.001	
	Americas	17 (959, 2465)	0.271 (0.177, 0.391)	96.65%	p<0.001	p<0.001	
	NA	15 (2632, 9443)	0.337 (0.225, 0.471)	98.97%	p=0.018	p<0.001	
	Multiple continents	1 (9, 105)	0.086 (0.045, 0.157)	0.00%	p<0.001	p>0.999	
	Africa	9 (214, 885)	0.251 (0.210, 0.297)	45.33%	p<0.001	p=0.067	
	Oceania	2 (1298, 1584)	0.819 (0.800, 0.838)	0.00%	p<0.001	p>0.999	
**AST method**	MIC	137 (19077, 67654)	0.329 (0.293, 0.367)	98.56%	p<0.001	p<0.001	p=0.209
	NA	11 (68630, 285385)	0.348 (0.266, 0.440)	95.99%	p=0.001	p<0.001	
	Disk Diffusion	22 (3203, 8609)	0.287 (0.234, 0.347)	95.81%	p<0.001	p<0.001	
	Multiple Method	7 (894, 1453)	0.438 (0.196, 0.713)	98.57%	p=0.673	p<0.001	
	Automate	1 (290, 413)	0.702 (0.656, 0.744)	0.00%	p<0.001	p>0.999	
**AST Guideline**	CLSI	48 (7122, 25412)	0.315 (0.264, 0.372)	98.21%	p<0.001	p<0.001	p=0.652
	EUCAST	87 (12293, 41067)	0.351 (0.304, 0.401)	98.60%	p<0.001	p<0.001	
	NA	41 (72397, 296356)	0.306 (0.251, 0.367)	98.38%	p<0.001	p<0.001	
	BSAC	1 (250, 540)	0.463 (0.421, 0.505)	0.00%	p=0.085	p>0.999	
	French microbiology society	1 (32, 139)	0.230 (0.168, 0.307)	0.00%	p<0.001	p>0.999	
**year group**	2015_2019	76 (10830, 36303)	0.291 (0.245, 0.341)	98.59%	p<0.001	p<0.001	p=0.012
	2020_2023	102 (81264, 327211)	0.365 (0.332, 0.399)	98.52%	p<0.001	p<0.001	
**quality. group**	Low Risk	167 (22703, 74402)	0.329 (0.296, 0.363)	98.49%	p<0.001	p<0.001	p=0.767

	Some Risk	11 (69391, 289112)	0.322 (0.267, 0.382)	96.46%	p<0.001	p<0.001	
**Azithromycin**							
**Overall**	NA	4 (730, 2514)	0.344 (0.175, 0.564)	0.00%	p<0.001	p=0.938	NA

Caption; K: Number of reports, n: Number of resistant isolates, N: Number of total isolates, LCI: 95% Lower Limit Confidence Interval, HCI: 95% Higher Limit Confidence Interval, P1: P-value of difference from zero resistance rate, P2: P-value of heterogeneity between reports, P3: P-value of difference between groups.

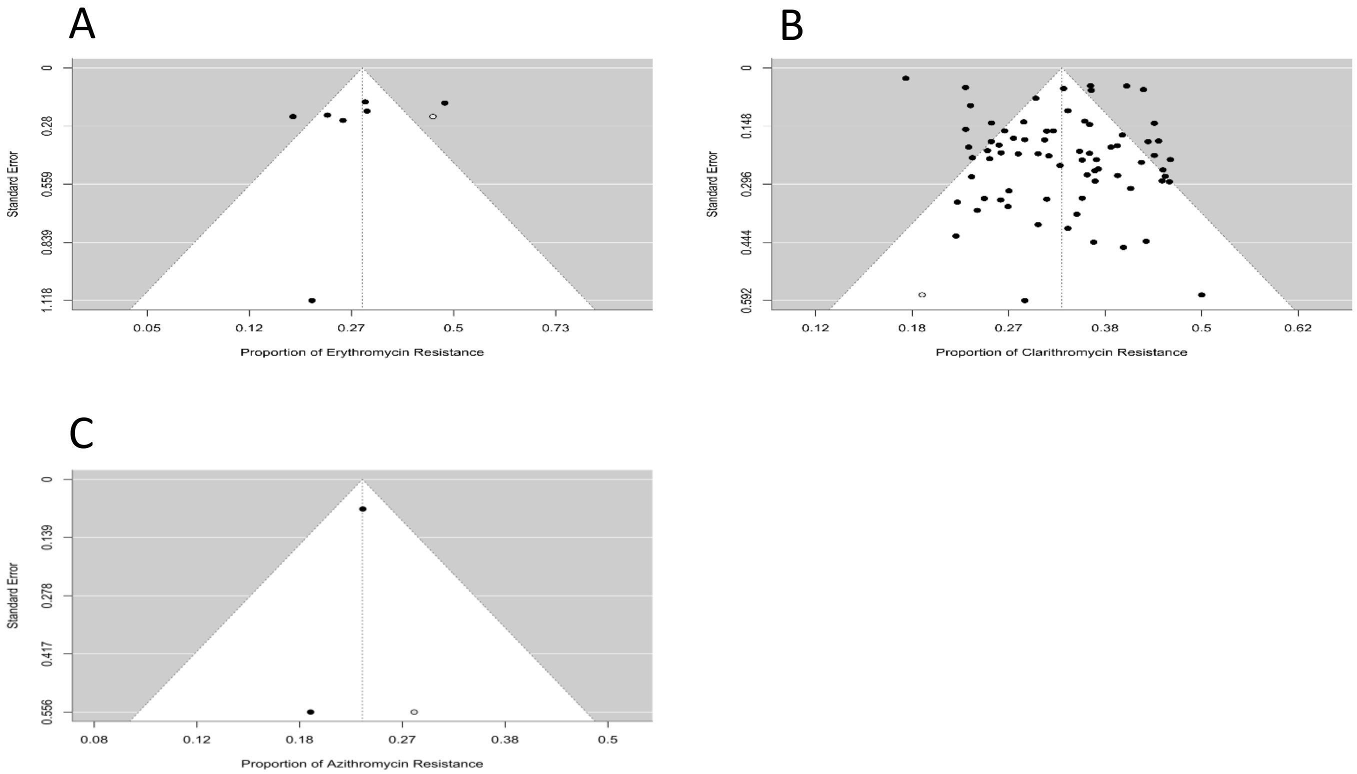

Figure. 2: **Funnel plots for investigation of publication bias**; (A) the funnel plot of Erythromycin -resistant *H. pylori* prevalence did not have significant evidence of publication bias; (B) the funnel plot of Clarythromycin-resistant *H. pylori* prevalence did not have significant evidence of publication bias; (C) The funnel plot of Azithromycin-resistant *H. pylori* prevalence did not have significant evidence of publication bias.

Abdollahi, H., M. Hashemzadeh, S. Khoshnood and M. Savari (2019). "Characterization of Helicobacter pylori genotypes from Iranian patients with gastric clinical diseases: Predominance of vacA s1a and cagA EPIYA-ABC genotypes." Gene Reports **16**: 100458.

Aftab, H., M. Miftahussurur, P. Subsomwong, F. Ahmed, A. A. Khan and Y. Yamaoka (2016). "Helicobacter pylori antibiotic susceptibility patterns in Bangladesh: Emerging levofloxacin resistance." The Journal of Infection in Developing Countries **10**(03): 245-253.

Akhtereeva, A., L. Morozova, R. Faizullina, K. Ivanovskaya, O. Pozdeev, I. K. Valeeva and S. Abdulkhakov (2018). "Antibiotic susceptibility assessment of Helicobacter pylori isolates by disk-diffusion method." BioNanoScience **8**(3): 930-934.

Alarcón, T., P. Urruzuno, M. J. Martínez, D. Domingo, L. Llorca, A. Correa and M. López-Brea (2017). "Antimicrobial susceptibility of 6 antimicrobial agents in Helicobacter pylori clinical isolates by using EUCAST breakpoints compared with previously used breakpoints." Enfermedades infecciosas y microbiologia clinica (English ed.) **35**(5): 278-282.

Alarcón-Millán, J., G. Fernández-Tilapa, E. M. Cortés-Malagón, C. A. Castañón-Sánchez, J. De Sampedro-Reyes, I. Cruz-del Carmen, R. Betancourt-Linares and A. Román-Román (2016). "Clarithromycin resistance and prevalence of Helicobacter pylori virulent genotypes in patients from Southern México with chronic gastritis." Infection, Genetics and Evolution **44**: 190-198.

Alavifard, H., N. Mirzaei, A. Yadegar, K. Baghaei, S. M. Smith, A. Sadeghi and M. R. Zali (2021). "Investigation of clarithromycin resistance-associated mutations and virulence genotypes of Helicobacter pylori isolated from Iranian population: a cross-sectional study." Current Microbiology **78**: 244-254.

Amin, M., A. A. Shayesteh, A. Serajian and H. Goodarzi (2019). "Assessment of Metronidazole and Clarithromycin Resistance Among Helicobacter pylori Isolates of Ahvaz (Southwest of Iran) During 2015 - 2016 by Phenotypic and Molecular Methods." Jundishapur J Microbiol **12**(4): e80156.

Ang, T. L., K. M. Fock, D. Ang, A. B. E. Kwek, E. K. Teo and S. Dhamodaran (2016). "The changing profile of Helicobacter pylori antibiotic resistance in Singapore: a 15-year study." Helicobacter **21**(4): 261-265.

Anis, S., S. R. Farooqi and S. K. Niaz (2021). "Characterization of domain V mutations in clinical isolates of Helicobacter pylori in Pakistan and their effect on clarithromycin MIC." Infection and Drug Resistance: 3393-3403.

Antunes, R., M. Oleastro, J. P. Nogueira and A. I. Lopes (2023). "Time trend prevalence of helicobacter pylori infection and endoscopic findings in symptomatic children in Portugal: A retrospective study based on three time points in 2009, 2014, and 2019." Helicobacter **28**(4): e12963.

Aumpan, N., R.-K. Vilaichone, P. Gumnarai, L. Sanglutong, T. Ratanachu-Ek, V. Mahachai and Y. Yamaoka (2020). "Prevalence and antibiotic resistance patterns of Helicobacter pylori infection in Koh Kong, Cambodia." Asian Pacific journal of cancer prevention: APJCP **21**(5): 1409.

Azrad, M., D. Vazana, A. On, M. Paritski, H. Rohana, H. Roshrosh, K. Agay-Shay and A. Peretz (2022). "Antibiotic resistance patterns of Helicobacter pylori in North Israel–A six-year study." Helicobacter **27**(6): e12932.

Bachir, M., R. Allem, L. Benejat, A. Tifrit, M. Medjekane, A. E.-M. Drici, F. Megraud and K. T. Douidi (2018). "Molecular detection of mutations involved in Helicobacter pylori antibiotic resistance in Algeria." Journal of Antimicrobial Chemotherapy **73**(8): 2034-2038.

Bachir, M., R. Allem, A. Tifrit, M. Medjekane, A. E.-M. Drici, M. Diaf and K. T. Douidi (2018). "Primary antibiotic resistance and its relationship with cagA and vacA genes in Helicobacter pylori isolates from Algerian patients." brazilian journal of microbiology **49**: 544-551.

Bahmaninejad, P., S. Ghafourian, M. Mahmoudi, A. Maleki, N. Sadeghifard and B. Badakhsh (2021). "Persister cells as a possible cause of antibiotic therapy failure in Helicobacter pylori." Jgh Open **5**(4): 493-497.
Bai, P., L. Y. Zhou, X. M. Xiao, Y. Luo and Y. Ding (2015). "Susceptibility of H elicobacter pylori to antibiotics in C hinese patients." Journal of Digestive Diseases **16**(8): 464-470.
Bang, C. S., H. Lim, H. M. Jeong, W. G. Shin, J. H. Choi, J. S. Soh, H. S. Kang, Y. J. Yang, J. T. Hong and S. P. Shin (2020). "Amoxicillin or tetracycline in bismuth-containing quadruple therapy as first-line treatment for Helicobacter pylori infection." Gut Microbes **11**(5): 1314-1323.
Bayati, S., M. Alebouyeh, N. Amirmozafari, N. Ebrahimi Daryani, M. Talebi and M. R. Zali (2020). "Histological changes in refractory Helicobacter pylori infection and its relationship with increased levels of resistance to antibiotics and therapeutic regimens: one-year follow-up." Apmis **128**(1): 25-34.
Bińkowska, A., M. M. Biernat, Ł. Łaczmański and G. Gościniak (2018). "Molecular patterns of resistance among Helicobacter pylori strains in south-western Poland." Frontiers in microbiology **9**: 410371.
Binyamin, D., N. Pastukh, A. On, M. Paritsky and A. Peretz (2017). "Phenotypic and genotypic correlation as expressed in Helicobacter pylori resistance to clarithromycin and fluoroquinolones." Gut Pathogens **9**: 1-8.
Boltin, D., H. Ben-Zvi, T. T. Perets, R. Gingold-Belfer, R. Dickman and Y. Niv (2015). "Appropriateness of Repeating Helicobacter pylori Culture and Susceptibility Testing Following Failure of Individualized Antibiotic Therapy." Digestion **92**(2): 66-72.
Boltin, D., H. Ben-Zvi, T. T. Perets, Z. Kamenetsky, Z. Samra, R. Dickman and Y. Niv (2015). "Trends in secondary antibiotic resistance of Helicobacter pylori from 2007 to 2014: has the tide turned?" Journal of clinical microbiology **53**(2): 522-527.
Boyanova, L., G. Gergova, R. Markovska, N. Kandilarov, L. Davidkov, Z. Spassova and I. Mitov (2017). "Primary Helicobacter pylori resistance in elderly patients over 20 years: A Bulgarian study." Diagnostic microbiology and infectious disease **88**(3): 264-267.
Bruce, M. G., D. Bruden, D. Newbrough, D. A. Hurlburt, T. W. Hennessy, J. M. Morris, A. L. Reasonover, F. Sacco and B. J. McMahon (2019). "The relationship between previous antimicrobial use, antimicrobial resistance and treatment outcome among Alaskans treated for Helicobacter pylori infection." GastroHep **1**(4): 172-179.
Bujanda, L., O. P. Nyssen, D. Vaira, I. M. Saracino, G. Fiorini, F. Lerang, S. Georgopoulos, B. Tepes, F. Heluwaert and A. Gasbarrini (2021). "Antibiotic resistance prevalence and trends in patients infected with Helicobacter pylori in the period 2013–2020: Results of the European Registry on H. pylori Management (Hp-EuReg)." Antibiotics **10**(9): 1058.
Buran, T., S. Sürücüoğlu, S. Kurutepe and H. Gazi (2022). "Recent trends in the antibiotic resistance of Helicobacter pylori in patient with dyspepsia." Medicine **101**(26): e29801.
Burayzat, S., M. Al-Tamimi, M. Barqawi, M. S. Massadi, J. Abu-Raideh, H. Albalawi, A. I. Khasawneh, N. Himsawi and M. Barber (2023). "Antimicrobial Resistance Molecular Mechanisms of Helicobacter pylori in Jordanian Children: A Cross-Sectional Observational Study." Antibiotics **12**(3): 618.
Butenko, T., S. Jeverica, R. Orel and M. Homan (2017). "Antibacterial resistance and the success of tailored triple therapy in Helicobacter pylori strains isolated from Slovenian children." Helicobacter **22**(5): e12400.
Caliskan, R., H. B. Tokman, Y. Erzin, S. Saribas, P. Yuksel, B. K. Bolek, E. O. Sevuk, M. Demirci, O. Yılmazli and O. Akgul (2015). "Antimicrobial resistance of Helicobacter pylori strains to five

antibiotics, including levofloxacin, in Northwestern Turkey." Revista da Sociedade Brasileira de Medicina Tropical **48**: 278-284.

Camorlinga-Ponce, M., A. Gómez-Delgado, E. Aguilar-Zamora, R. C. Torres, S. Giono-Cerezo, A. Escobar-Ogaz and J. Torres (2021). "Phenotypic and genotypic antibiotic resistance patterns in Helicobacter pylori strains from ethnically diverse population in Mexico." Frontiers in cellular and infection microbiology **10**: 539115.

Chen, D., S. A. Cunningham, N. C. Cole, P. C. Kohner, J. N. Mandrekar and R. Patel (2017). "Phenotypic and molecular antimicrobial susceptibility of Helicobacter pylori." Antimicrobial agents and chemotherapy **61**(4): 10.1128/aac. 02530-02516.

Chen, J., Y. Huang, Z. Ding, X. Liang and H. Lu (2022). "E-test or agar dilution for metronidazole susceptibility testing of Helicobacter pylori: importance of the prevalence of metronidazole resistance." Frontiers in Microbiology **13**: 801537.

Choi, Y. I., S.-H. Jeong, J.-W. Chung, D. K. Park, K. O. Kim, K. A. Kwon, Y. J. Kim, S. So, J. H. Lee and J.-Y. Jeong (2019). "Rifabutin and furazolidone could be the candidates of the rescue regimen for antibiotic-resistant H. pylori in Korea." Canadian Journal of Infectious Diseases and Medical Microbiology **2019**.

Choi, Y. I., S. M. Lee, J.-W. Chung, K. O. Kim, K. A. Kwon, Y. J. Kim, J. H. Kim, S. M. Lee, J.-Y. Jeong and D. K. Park (2021). "Therapeutic Potential of Sitafloxacin as a New Drug Candidate for Helicobacter Eradication in Korea: An In Vitro Culture-Based Study." Antibiotics **10**(10): 1242.

Cosme, A., M. Montes, B. Ibarra, E. Tamayo, H. Alonso, U. Mendarte, J. Lizasoan, M. Herreros-Villanueva and L. Bujanda (2017). "Antimicrobial susceptibility testing before first-line treatment for Helicobacter pylori infection in patients with dual or triple antibiotic resistance." World J Gastroenterol **23**(18): 3367-3373.

Cosme, A., S. Torrente Iranzo, M. Montes Ros, M. Fernández-Reyes Silvestre, H. Alonso Galan, J. Lizasoain and L. Bujanda (2019). "Helicobacter pylori antimicrobial resistance during a 5-year period (2013-2017) in northern Spain and its relationship with the eradication therapies." Helicobacter **24**(1): e12557.

Dai, J., J. Zhao, L. Mao, Y. Hu and B. Lv (2022). "Study on the value of antibiotic-resistant gene detection in Helicobacter pylori in China." Experimental and Therapeutic Medicine **23**(3): 1-10.

de Arbulo, M. G.-R., E. Tamayo, L. Bujanda, L. Mendibil, J. Mendiola, G. Cilla and M. Montes (2023). "Surveillance of Helicobacter pylori resistance over 22 Years (2000-2021) in Northern Spain." Journal of Global Antimicrobial Resistance **34**: 127-133.

Deng, R., L. Liu, W. Xie, W. Lu, Z. Liu and Y. Wang (2023). "Prevalence of Helicobacter pylori Antibiotic Resistance in Patients Enrolled in Guangzhou, China." Infection and Drug Resistance: 5033-5038.

Di Giulio, M., E. Di Campli, S. Di Bartolomeo, V. Cataldi, L. Marzio, L. Grossi, A. F. Ciccaglione, A. Nostro and L. Cellini (2016). "In vitro antimicrobial susceptibility of Helicobacter pylori to nine antibiotics currently used in Central Italy." Scandinavian journal of gastroenterology **51**(3): 263-269.

Eed, E. M., Y. A. Hawash, A. S. Khalifa, K. F. Alsharif, S. A. Alghamdi, T. Saber, K. A. Ismail and S. A. Shehab-Eldeen (2019). "Molecular diagnosis of Helicobacter pylori antibiotic resistance in the Taif region, Saudi Arabia." Microbiology and immunology **63**(6): 199-205.

Eng, N. F., G. Ybazeta, K. Chapman, N. L. Fraleigh, R. Letto, E. Altman and F. Diaz-Mitoma (2015). "Antimicrobial susceptibility of Canadian isolates of Helicobacter pylori in Northeastern Ontario." Can J Infect Dis Med Microbiol **26**(3): 137-144.

Erkut, M., D. Y. Uzun, N. Kaklıkkaya, S. Fidan, Y. Yoğun, A. M. Coşar, E. Akyıldız, M. Topbaş, O. Özgür and M. Arslan (2020). "Sociodemographic characteristics and clinical risk factors of Helicobacter pylori infection and antibiotic resistance in the Eastern Black Sea region of Turkey." The Turkish Journal of Gastroenterology **31**(3): 221.
Farzi, N., C. Behzad, Z. Hasani, M. Alebouyeh, H. Zojaji and M. R. Zali (2019). "Characterization of clarithromycin heteroresistance among Helicobacter pylori strains isolated from the antrum and corpus of the stomach." Folia microbiologica **64**: 143-151.
Fauzia, K. A., H. Aftab, E. Tshibangu-Kabamba, R. I. Alfaray, B. Saruuljavkhlan, A. Cimuanga-Mukanya, T. Matsumoto, P. Subsomwong, J. Akada and M. Miftahussurur (2023). "Mutations related to antibiotics resistance in Helicobacter pylori clinical isolates from Bangladesh." Antibiotics **12**(2): 279.
Fauzia, K. A., M. Miftahussurur, A. F. Syam, L. A. Waskito, D. Doohan, Y. A. A. Rezkitha, T. Matsumoto, V. P. Tuan, J. Akada and H. Yonezawa (2020). "Biofilm formation and antibiotic resistance phenotype of Helicobacter pylori clinical isolates." Toxins **12**(8): 473.
Fiorini, G., I. M. Saracino, A. Zullo, L. Gatta, M. Pavoni and D. Vaira (2017). "Rescue therapy with bismuth quadruple regimen in patients with Helicobacter pylori-resistant strains." Helicobacter **22**(6): e12448.
Fiorini, G., A. Zullo, I. M. Saracino, L. Gatta, M. Pavoni and D. Vaira (2018). "Pylera and sequential therapy for first-line Helicobacter pylori eradication: a culture-based study in real clinical practice." European Journal of Gastroenterology & Hepatology **30**(6): 621-625.
Fiorini, G., A. Zullo, I. M. Saracino, M. Pavoni and D. Vaira (2018). "Antibiotic resistance pattern of Helicobacter pylori strains isolated in Italy during 2010–2016." Scandinavian Journal of Gastroenterology **53**(6): 661-664.
Gatta, L., C. Scarpignato, G. Fiorini, J. Belsey, I. Saracino, C. Ricci and D. Vaira (2018). "Impact of primary antibiotic resistance on the effectiveness of sequential therapy for Helicobacter pylori infection: lessons from a 5-year study on a large number of strains." Alimentary Pharmacology & Therapeutics **47**(9): 1261-1269.
Gehlot, V., S. Mahant, A. K. Mukhopadhyay, K. Das, J. Alam, P. Ghosh and R. Das (2016). "Low prevalence of clarithromycin-resistant Helicobacter pylori isolates with A2143G point mutation in the 23S rRNA gene in North India." Journal of Global Antimicrobial Resistance **6**: 39-43.
Gehlot, V., S. Mahant, A. K. Mukhopadhyay, K. Das, R. De, P. Kar and R. Das (2016). "Antimicrobial susceptibility profiles of Helicobacter pylori isolated from patients in North India." J Glob Antimicrob Resist **5**: 51-56.
Goni, E., I. Tammer, K. Schütte, C. Thon, D. Jechorek, U. M. Mahajan, R. Vasapolli, L. Macke, B. Aulinger and M. Selgrad (2022). "The influence of gastric atrophy on Helicobacter pylori antibiotics resistance in therapy-naïve patients." Frontiers in Microbiology **13**: 938676.
Gonzalez-Hormazabal, P., M. Musleh, S. Escandar, H. Valladares, E. Lanzarini, V. G. Castro, L. Jara and Z. Berger (2018). "Prevalence of clarithromycin resistance in Helicobacter pylori in Santiago, Chile, estimated by real-time PCR directly from gastric mucosa." BMC gastroenterology **18**: 1-5.
Gunnarsdottir, A. I., H. Gudjonsson, H. Hardardottir, K. D. Jonsdottir and E. S. Bjornsson (2017). "Antibiotic susceptibility of Helicobacter pylori in Iceland." Infectious Diseases **49**(9): 647-654.
Guo, C., F. Liu, L. Zhu, F. Wu, G. Cui, Y. Xiong, Q. Wang, L. Yin, C. Wang and H. Wang (2019). "Analysis of culturable microbiota present in the stomach of children with gastric symptoms." Brazilian Journal of Microbiology **50**: 107-115.

Hallur, V., M. Panigrahi, M. Sable, M. Ghosh, S. Mohanty, S. Purkait and A. Praharaj (2022). "Low clarithromycin resistance in virulent Helicobacter pylori from dyspeptic patients at a tertiary care centre in Odisha." Indian Journal of Medical Microbiology **40**(2): 211-216.
Hamidi, S., F. Badmasti, F. Sadeghpour Heravi, M. H. Safapoor, A. Mohammad Ali Tabrizi, M. Ghorbani and O. Azizi (2020). "Antibiotic resistance and clonal relatedness of Helicobacter pylori strains isolated from stomach biopsy specimens in northeast of Iran." Helicobacter **25**(2): e12684.
Hanafiah, A., H. Binmaeil, R. A. Raja Ali, I. Mohamed Rose and B. S. Lopes (2019). "Molecular characterization and prevalence of antibiotic resistance in Helicobacter pylori isolates in Kuala Lumpur, Malaysia." Infection and drug resistance: 3051-3061.
Hanafy, A. S. and W. M. Seleem (2019). "Refractory Helicobacter pylori gastritis: The hidden predictors of resistance." Journal of Global Antimicrobial Resistance **19**: 194-200.
Hays, C., T. Delerue, D. Lamarque, C. Burucoa, G. Collobert, A. Billöet, N. Kalach and J. Raymond (2019). "Molecular diagnosis of Helicobacter pylori infection in gastric biopsies: Evaluation of the Amplidiag® H. pylori+ ClariR assay." Helicobacter **24**(2): e12560.
Helmbold, L., B. Ghebremedhin, A. Bellm, M. A. Hopkins, S. Wirth and M. Aydin (2022). "Increased antibiotic resistance in children with Helicobacter pylori infection: a retrospective study." Pathogens **11**(2): 178.
Hu, L., X. Zeng, Q. Ai, C. Liu, X. Zhang, Y. Chen, L. Liu and G.-Q. Li (2023). "Long-read-and short-read-based whole-genome sequencing reveals the antibiotic resistance pattern of Helicobacter pylori." Microbiology Spectrum **11**(3): e04522-04522.
Hu, S., Y. Zhou, Y. Deng, Y. Bo, X. Chen, W. Yang, R. Shi, W. Zhao, Z. Hou and J. Hu (2023). "Characteristics of phenotypic antibiotic resistance of Helicobacter pylori and its correlation with genotypic antibiotic resistance: A retrospective study in Ningxia." Helicobacter **28**(3): e12960.
Huang, X., B. Wu, Q. Chen, Y. Chen, X. Ji, X. Zhou, B. Suo, Z. Lin and X. Zheng (2023). "Antibiotic resistance profile of Helicobacter pylori to 14 antibiotics: a multicenter study in Fujian, China." PeerJ **11**: e15611.
IM, S. (2020). "Antibiotic resistance and therapy outcome in H. pylori eradication failure patients." Antibiotics **9**(3): 121.
Ji, Z., F. Han, F. Meng, M. Tu, N. Yang and J. Zhang (2016). "The association of age and antibiotic resistance of Helicobacter pylori: a study in Jiaxing City, Zhejiang Province, China." Medicine **95**(8): e2831.
Jiang, Z., X. Qian, Z. Wang, Y. Dong, Y. Pan, Z. Zhang and S. Wang (2022). "Antibiotic resistance of Helicobacter pylori isolated from patients in Nanjing, China: A cross-section study from 2018 to 2021." Frontiers in cellular and infection microbiology **12**: 970630.
Kageyama, C., M. Sato, H. Sakae, Y. Obayashi, Y. Kawahara, T. Mima, O. Matsushita, K. Yokota, M. Mizuno and H. Okada (2019). "Increase in antibiotic resistant Helicobacter pylori in a University Hospital in Japan." Infection and drug resistance: 597-602.
Khani, S., A. Talebi Bezmin Abadi and A. Mohabati Mobarez (2019). "Clarithromycin-susceptible but virulent Helicobacter pylori strains infecting Iranian patients' stomachs." Infection and Drug Resistance: 3415-3420.
Khoury, J., Y. Geffen, R. Shaul, H. Sholy, Y. Chowers and T. Saadi (2017). "Secondary antibiotic resistance of Helicobacter pylori isolates in Israeli children and adults." Journal of global antimicrobial resistance **10**: 182-185.
Kim, Y. M., K. H. Lee, J.-H. Kim, S. Y. Park, Y. G. Song, S. Y. Jeon and H. Park (2020). "Is only clarithromycin susceptibility important for the successful eradication of Helicobacter pylori?" Antibiotics **9**(9): 589.

Kobatake, T., K. Ogino, H. Sakae, K. Gotoh, A. Watanabe, O. Matsushita, H. Okada and K. Yokota (2021). "Antibacterial effects of disulfiram in Helicobacter pylori." Infection and Drug Resistance: 1757-1764.
Korona-Glowniak, I., H. Cichoz-Lach, R. Siwiec, S. Andrzejczuk, A. Glowniak, P. Matras and A. Malm (2019). "Antibiotic resistance and genotypes of Helicobacter pylori strains in patients with gastroduodenal disease in Southeast Poland." Journal of clinical medicine **8**(7): 1071.
Kouitcheu Mabeku, L. B., B. Eyoum Bille, C. Tepap Zemnou, L. D. Tali Nguefack and H. Leundji (2019). "Broad spectrum resistance in Helicobacter pylori isolated from gastric biopsies of patients with dyspepsia in Cameroon and efflux-mediated multiresistance detection in MDR isolates." BMC infectious diseases **19**: 1-11.
Kuo, C.-J., C.-H. Lee, M.-L. Chang, C.-Y. Lin, W.-R. Lin, M.-Y. Su, C.-H. Chiu, C.-N. Tseng, Y.-S. Wu and C.-T. Chiu (2021). "Multidrug resistance: the clinical dilemma of refractory Helicobacter pylori infection." Journal of Microbiology, Immunology and Infection **54**(6): 1184-1187.
Le Thi, T. G., K. Werkstetter, K. Kotilea, P. Bontems, J. Cabral, M. L. Cilleruelo Pascual, M. Kori, J. Barrio, M. Homan and N. Kalach (2023). "Management of Helicobacter pylori infection in paediatric patients in Europe: results from the EuroPedHp Registry." Infection **51**(4): 921-934.
Lee, J. H., J. Y. Ahn, K. D. Choi, H. Y. Jung, J. M. Kim, G. H. Baik, B. W. Kim, J. C. Park, H. K. Jung and S. J. Cho (2019). "Nationwide antibiotic resistance mapping of Helicobacter pylori in Korea: a prospective multicenter study." Helicobacter **24**(4): e12592.
Lee, J. W., N. Kim, R. H. Nam, S. M. Lee, C. Soo In, J. M. Kim and D. H. Lee (2019). "Risk factors of rescue bismuth quadruple therapy failure for Helicobacter pylori eradication." Journal of Gastroenterology and Hepatology **34**(4): 666-672.
Lee, K. H., S. Y. Park, S. J. Jeong, J.-H. Kim, S. H. Jeong, I.-M. Kang and Y. G. Song (2019). "Can aminoglycosides be used as a new treatment for Helicobacter pylori? In vitro activity of recently isolated Helicobacter pylori." Infection & Chemotherapy **51**(1): 10.
Li, J., J. Deng, Z. Wang, H. Li and C. Wan (2021). "Antibiotic resistance of Helicobacter pylori strains isolated from pediatric patients in Southwest China." Frontiers in Microbiology **11**: 621791.
Li, L., Y. Ke, C. Yu, G. Li, N. Yang, J. Zhang and Y. Li (2017). "Antibiotic resistance of Helicobacter pylori in Chinese children: a multicenter retrospective study over 7 years." Helicobacter **22**(3): e12373.
Li, L., W. Zhou, H. Li, C. Yu, T. Yan, N. Yang and Y.-M. Li (2021). "Antibiotic resistance of Helicobacter pylori isolated from patients after partial gastrectomy: A retrospective study." The Turkish Journal of Gastroenterology **32**(12): 996.
Li, Y., X. Li and Z. Tan (2021). "An overview of traditional Chinese medicine therapy for Helicobacter pylori–related gastritis." Helicobacter **26**(3): e12799.
Liang, C.-M., W.-C. Tai, P.-I. Hsu, D.-C. Wu, C.-H. Kuo, F.-W. Tsay, C.-L. Lee, K.-Y. Chen and S.-K. Chuah (2020). "Trend of changes in antibiotic resistance in Helicobacter pylori from 2013 to 2019: a multicentre report from Taiwan." Therapeutic Advances in Gastroenterology **13**: 1756284820976990.
Liu, D.-S., Y.-H. Wang, Z.-R. Zeng, Z.-Y. Zhang, H. Lu, J.-M. Xu, Y.-Q. Du, Y. Li, J.-B. Wang and S.-P. Xu (2018). "Primary antibiotic resistance of Helicobacter pylori in Chinese patients: a multiregion prospective 7-year study." Clinical microbiology and infection **24**(7): 780. e785-780. e788.
Liu, J., C.-R. Ji, Y.-Y. Li, C. Qiao, J.-N. Hu, M. Wan, M.-J. Lin, B.-S. Lin, J. Wang and J. Zha (2021). "Two different 1-week quadruple therapies given back-to-back consecutive therapy for

difficult-to-treat Helicobacter pylori infection: a pilot study." Clinical and Translational Gastroenterology **12**(8): e00391.
Liu, Y., S. Wang, F. Yang, W. Chi, L. Ding, T. Liu, F. Zhu, D. Ji, J. Zhou and Y. Fang (2022). "Antimicrobial resistance patterns and genetic elements associated with the antibiotic resistance of Helicobacter pylori strains from Shanghai." Gut pathogens **14**(1): 14.
Lok, C.-H., D. Zhu, J. Wang, Y.-T. Ren, X. Jiang, S.-J. Li and X.-Y. Zhao (2020). "Phenotype and molecular detection of clarithromycin and levofloxacin resistance in Helicobacter pylori clinical isolates in Beijing." Infection and Drug Resistance: 2145-2153.
Long, X., Q. Chen, L. Yu, X. Liang, W. Liu and H. Lu (2018). "Bismuth improves efficacy of proton-pump inhibitor clarithromycin, metronidazole triple Helicobacter pylori therapy despite a high prevalence of antimicrobial resistance." Helicobacter **23**(3): e12485.
Lu, H. H., F. P. Lai, H. Y. Lo, B. S. Sheu and Y. J. Yang (2019). "Increasing antimicrobial resistance to clarithromycin and metronidazole in pediatric Helicobacter pylori infection in southern Taiwan: A comparison between two decades." Helicobacter **24**(5): e12633.
Mahmoudi, S., S. Mamishi, M. Banar, S. K. Valian, A. Bahador, M. Najafi, F. Farahmand and B. Pourakbari (2017). "Antibiotic susceptibility of Helicobacter pylori strains isolated from Iranian children: high frequency of A2143G point mutation associated with clarithromycin resistance." Journal of global antimicrobial resistance **10**: 131-135.
Maleknejad, S., A. Mojtahedi, A. Safaei-Asl, Z. Taghavi and E. Kazemnejad (2015). "Primary antibiotic resistance to Helicobacter pylori strains isolated from children in Northern Iran: a single center study." Iranian journal of pediatrics **25**(6).
Manfredi, M., P. Gismondi, V. Maffini, B. Bizzarri, F. Fornaroli, C. Madia, A. Salerno, A. M. Cangelosi and G. L. de'Angelis (2015). "Primary antimicrobial susceptibility changes in children with Helicobacter pylori infection over 13 years in Northern Italy." Gastroenterology Research and Practice **2015**.
Mansour, K. B., C. Fendri, H. Battikh, M. Garnier, M. Zribi, A. Jlizi and C. Burucoa (2016). "Multiple and mixed Helicobacter pylori infections: comparison of two epidemiological situations in Tunisia and France." Infection, Genetics and Evolution **37**: 43-48.
Mascellino, M. T., A. Oliva, M. C. Miele, M. De Angelis, G. Bruno and C. Severi (2020). "Secondary antibiotic resistance, correlation between genotypic and phenotypic methods and treatment in Helicobacter pylori infected patients: a retrospective study." Antibiotics **9**(9): 549.
Matta, A. J., D. C. Zambrano and A. J. Pazos (2018). "Punctual mutations in 23S rRNA gene of clarithromycin-resistant Helicobacter pylori in Colombian populations." World Journal of Gastroenterology **24**(14): 1531.
Miftahussurur, M., M. Cruz, P. Subsomwong, J. A. J. Abreu, C. Hosking, H. Nagashima, J. Akada and Y. Yamaoka (2017). "Clarithromycin-based triple therapy is still useful as an initial treatment for Helicobacter pylori infection in the Dominican Republic." The American journal of tropical medicine and hygiene **96**(5): 1050.
Morimoto, N., H. Takeuchi, Y. Nishida, M. Morisawa, T. Yoshikawa, T. Morita, M. Morimoto, C. Sugimoto, Y. Matsumura and T. Sugiura (2015). "Clinical Application of the DiversiLab Microbial Typing System Using Repetitive Sequence-Based PCR for Characterization of Helicobacter pylori in Japan." Journal of clinical laboratory analysis **29**(3): 250-253.
Mormeneo Bayo, S., A. Bellés Bellés, D. Vázquez Gómez, M. Planella de Rubinat, D. C. Bayas Pastor, A. Morales Portillo, A. Jover Sáenz, É. López González, N. Prim and M. García-González (2023). "Antibiotic Susceptibility and Clarithromycin Resistance Determinants in Helicobacter pylori in the Northeast of Spain: A One-Year Prospective Study." Antibiotics **12**(2): 356.

Mosites, E., D. Bruden, J. Morris, A. Reasonover, K. Rudolph, D. Hurlburt, T. Hennessy, B. McMahon and M. Bruce (2018). "Antimicrobial resistance among Helicobacter pylori isolates in Alaska, 2000–2016." Journal of global antimicrobial resistance **15**: 148-153.
Nahm, J. H., W. K. Kim, Y. Kwon and H. Kim (2019). "Detection of Helicobacter pylori with clarithromycin resistance-associated mutations using peptide nucleic acid probe-based melting point analysis." Helicobacter **24**(5): e12634.
Nguyen, T. C., G. K. N. Le, D. T. H. Pham, B. V. Pham, L. T. H. Nguyen, T. H. Che, H. T. Nguyen, D. Q. Truong, A. Robert and P. Bontems (2023). "Antibiotic resistance and heteroresistance in Helicobacter pylori isolates from symptomatic Vietnamese children: a prospective multicenter study." Helicobacter **28**(5): e13009.
Oporto, M., M. Pavez, C. Troncoso, A. Cerda, E. Hofmann, A. Sierralta, E. Rios, L. Coppelli and L. Barrientos (2019). "Prevalence of infection and antibiotic susceptibility of Helicobacter pylori: an evaluation in public and private health systems of Southern Chile." Pathogens **8**(4): 226.
Örsten, S., E. Yılmaz and Y. Akyön (2023). "Molecular Characterization of Clarithromycin Resistance in Helicobacter pylori Strains." The Turkish Journal of Gastroenterology **34**(4): 427.
Peña, J., H. Rojas, N. Reyes, M. Fernández-Delgado, M.-A. García-Amado, F. Michelangeli and M. Contreras (2017). "Multiple cag genotypes of Helicobacter pylori isolates colonize the oesophagus in individual hosts in a Venezuelan population." Journal of Medical Microbiology **66**(2): 226-235.
Pichon, M., C. T. Tran, G. Motillon, C. Debiais, S. Gautier, M. Aballea, J. Cremniter, P. Vasseur, D. Tougeron and M. Garcia (2020). "Where to biopsy to detect helicobacter pylori and how many biopsies are needed to detect antibiotic resistance in a human stomach." Journal of Clinical Medicine **9**(9): 2812.
Quek, C., S. T. Pham, K. T. Tran, B. T. Pham, L. V. Huynh, N. B. Luu, T. K. Le, K. Quek and V. H. Pham (2016). "Antimicrobial susceptibility and clarithromycin resistance patterns of Helicobacter pylori clinical isolates in Vietnam." F1000Research **5**.
Raaf, N., W. Amhis, H. Saoula, A. Abid, M. Nakmouche, A. Balamane, N. Ali Arous, M. Ouar-Korichi, F. F. Vale and L. Bénéjat (2017). "Prevalence, antibiotic resistance, and MLST typing of Helicobacter pylori in Algiers, Algeria." Helicobacter **22**(6): e12446.
Rasi-Bonab, F., A. Jafari-Sales, M. A. Shaverdi, T. Navidifar, M. Saki, A. Ghorbani, A. O. Adekanmbi, B. Jafari and S. Naebi (2021). "Antibiotic resistance pattern and frequency of cagA and vacA genes in Helicobacter pylori strains isolated from patients in Tabriz city, Iran." BMC Research Notes **14**: 1-5.
Redondo, J. J., P. M. Keller, R. Zbinden and K. Wagner (2018). "A novel RT-PCR for the detection of Helicobacter pylori and identification of clarithromycin resistance mediated by mutations in the 23S rRNA gene." Diagnostic microbiology and infectious disease **90**(1): 1-6.
Regnath, T., O. Raecke, A. Enninger and R. Ignatius (2017). "Increasing metronidazole and rifampicin resistance of Helicobacter pylori isolates obtained from children and adolescents between 2002 and 2015 in southwest Germany." Helicobacter **22**(1): e12327.
Rezaei, S., A. T. B. Abadi and A. M. Mobarez (2020). "Metronidazole-resistant Helicobacter pylori isolates without rdxA mutations obtained from Iranian dyspeptic patients." New Microbes and New Infections **34**: 100636.
Sadeghi, H., T. Narimani, E. Tabesh, F. Shafiee and R. Soltani (2022). "Antibiotic resistance pattern of Helicobacter pylori strains isolated from patients in Isfahan, Iran." Journal of Research in Medical Sciences **27**(1): 39.

Salehi, N., B. Attaran, F. Zare-Mirakabad, B. Ghadiri, M. Esmaeili, M. Shakaram, M. Tashakoripour, M. Eshagh Hosseini and M. Mohammadi (2020). "The outward shift of clarithromycin binding to the ribosome in mutant Helicobacter pylori strains." Helicobacter **25**(6): e12731.
Saracino, I. M., G. Fiorini, A. Zullo, M. Pavoni, L. Saccomanno and D. Vaira (2020). "Trends in primary antibiotic resistance in H. pylori strains isolated in Italy between 2009 and 2019." Antibiotics **9**(1): 26.
Schubert, J. P., P. R. Ingram, M. S. Warner, C. K. Rayner, I. C. Roberts-Thomson, S. P. Costello and R. V. Bryant (2023). "Refractory Helicobacter pylori infection in Australia: updated multicentre antimicrobial resistance." Internal Medicine Journal **53**(11): 1972-1978.
Schwarzer, A., P. Bontems, P. Urruzuno, N. Kalach, B. Iwanczak, E. Roma-Giannikou, J. Sykora, A. Kindermann, T. Casswall and S. Cadranel (2016). "Sequential therapy for Helicobacter pylori infection in treatment-naive children." Helicobacter **21**(2): 106-113.
Seo, J. W., J. Y. Park, T.-S. Shin and J. G. Kim (2019). "The analysis of virulence factors and antibiotic resistance between Helicobacter pylori strains isolated from gastric antrum and body." BMC gastroenterology **19**: 1-8.
Shao, Y., R. Lu, Y. Yang, Q. Xu, B. Wang and G. Ye (2018). "Antibiotic resistance of Helicobacter pylori to 16 antibiotics in clinical patients." Journal of clinical laboratory analysis **32**(4): e22339.
Shiota, S., R. Reddy, A. Alsarraj, H. B. El-Serag and D. Y. Graham (2015). "Antibiotic resistance of Helicobacter pylori among male United States veterans." Clinical Gastroenterology and Hepatology **13**(9): 1616-1624.
Shokrzadeh, L., M. Alebouyeh, T. Mirzaei, N. Farzi and M. R. Zali (2015). "Prevalence of multiple drug-resistant Helicobacter pylori strains among patients with different gastric disorders in Iran." Microbial Drug Resistance **21**(1): 105-110.
Shoosanglertwijit, R., N. Kamrat, D. Werawatganon, T. Chatsuwan, S. Chaithongrat and R. Rerknimitr (2020). "Real-world data of Helicobacter pylori prevalence, eradication regimens, and antibiotic resistance in Thailand, 2013–2018." JGH Open **4**(1): 49-53.
Shu, X., D. Ye, C. Hu, K. Peng, H. Zhao, H. Li and M. Jiang (2022). "Alarming antibiotics resistance of Helicobacter pylori from children in Southeast China over 6 years." Scientific Reports **12**(1): 17754.
Shu, X., G. Yin, M. Liu, K. Peng, H. Zhao and M. Jiang (2018). "Antibiotics resistance of Helicobacter pylori in children with upper gastrointestinal symptoms in Hangzhou, China." Helicobacter **23**(3): e12481.
Song, Z., L. Zhou, J. Zhang, L. He, P. Bai and Y. Xue (2016). "Levofloxacin, bismuth, amoxicillin and esomeprazole as second-line Helicobacter pylori therapy after failure of non-bismuth quadruple therapy." Digestive and Liver Disease **48**(5): 506-511.
Sukri, A., A. Hanafiah, H. Yusoff, N. A. Shamsul Nizam, Z. Nameyrra, Z. Wong and R. A. Raja Ali (2022). "Multidrug-resistant Helicobacter pylori strains: a five-year surveillance study and its genome characteristics." Antibiotics **11**(10): 1391.
Taghvaei, T., N. Kamali, A. T. B. Abadi, F. Rahimi and M. Forootan (2021). "Isolation of dupA-positive and clarithromycin-resistant Helicobacter pylori from Iranian patients with duodenal ulcer." Gene Reports **24**: 101228.
Tamayo, E., M. Montes, M. Fernández-Reyes, J. Lizasoain, B. Ibarra, U. Mendarte, E. Zapata, J. Mendiola and E. Pérez-Trallero (2017). "Clarithromycin resistance in Helicobacter pylori and its molecular determinants in Northern Spain, 2013–2015." Journal of global antimicrobial resistance **9**: 43-46.

Tang, X., X. Chen, Y. Shen, T. Yang, R. Hu, A. W. Debowski, K. A. Stubbs, M. Benghezal, B. J. Marshall and H. Li (2020). "Primary antibiotic resistance of Helicobacter pylori among a Chinese Tibetan population." Future Microbiology **15**(14): 1353-1361.
Tang, X., Z. Wang, Y. Shen, X. Song, M. Benghezal, B. J. Marshall, H. Tang and H. Li (2022). "Antibiotic resistance patterns of Helicobacter pylori strains isolated from the Tibet Autonomous Region, China." BMC microbiology **22**(1): 196.
Tong, Y.-F., J. Lv, L.-Y. Ying, F. Xu, B. Qin, M.-T. Chen, F. Meng, M.-Y. Tu, N.-M. Yang and Y.-M. Li (2015). "Seven-day triple therapy is a better choice for Helicobacter pylori eradication in regions with low antibiotic resistance." World Journal of Gastroenterology **21**(46): 13073.
Vagarali, M., S. Metgud, H. Bannur, S. Karadesai and J. Nagmoti (2015). "Clinical significance of various diagnostic techniques and emerging antimicrobial resistance pattern of Helicobacter pylori from gastric biopsy samples." Indian journal of medical microbiology **33**(4): 560-564.
Van den Poel, B., S. Gils, I. Micalessi, S. Carton, P. Christiaens, P.-J. Cuyle, V. Moons, G. Van Olmen, A. Smismans and C. Bourgain (2021). "Molecular detection of Helicobacter pylori and clarithromycin resistance in gastric biopsies: a prospective evaluation of RIDA® GENE Helicobacter pylori assay." Acta Clinica Belgica **76**(3): 177-183.
Vazirzadeh, J., J. Falahi, S. Moghim, T. Narimani, R. Rafiei and V. Karbasizadeh (2020). "Molecular assessment of resistance to clarithromycin in Helicobacter pylori strains isolated from patients with dyspepsia by fluorescent in situ hybridization in the center of Iran." BioMed Research International **2020**.
Vilaichone, R.-k., N. Aumpan, T. Ratanachu-Ek, T. Uchida, L. Tshering, V. Mahachai and Y. Yamaoka (2020). "Population-based study of Helicobacter pylori infection and antibiotic resistance in Bhutan." International Journal of Infectious Diseases **97**: 102-107.
Vu, T. B., T. N. Q. Tran, T. Q. A. Tran, D. L. Vu and V. T. Hoang (2022). "Antibiotic resistance of Helicobacter pylori in patients with peptic ulcer." Medicina **59**(1): 6.
Wang, B., Q. Zhao, W. Yin, Y. Yuan, X. Wang, Y.-h. Wang, H. Wang, W. Ye, S. Chen and H.-l. Guo (2018). "In-vitro characterisation of a novel antimicrobial agent, TNP-2092, against Helicobacter pylori clinical isolates." Swiss Medical Weekly **148**(2930): w14630-w14630.
Wang, Y., Y. Li, Y. Gong, Y. Dong, J. Sun and M. Chen (2023). "Antibiotic resistance characteristics and risk factors analysis of Helicobacter pylori strains isolated from patients in Liaoning Province, an area in North China." PeerJ **11**: e15268.
Wang, Y. h., Z. f. Lv, Y. Zhong, D. s. Liu, S. p. Chen and Y. Xie (2017). "The internalization of Helicobacter pylori plays a role in the failure of H. pylori eradication." Helicobacter **22**(1): e12324.
Wani, F. A., G. Bashir, M. A. Khan, S. A. Zargar, Z. Rasool and Q. Qadri (2018). "Antibiotic resistance in Helicobacter pylori: a mutational analysis from a tertiary care hospital in Kashmir, India." Indian journal of medical microbiology **36**(2): 265-272.
Wu, M.-C., Y.-K. Wang, C.-J. Liu, F.-J. Yu, F.-C. Kuo, M.-L. Liu, C.-H. Kuo, D.-C. Wu, Y.-K. Huang and I.-C. Wu (2017). "Adding bismuth to rabeprazole-based first-line triple therapy does not improve the eradication of Helicobacter pylori." Gastroenterology Research and Practice **2017**.
Xiong, M., H. S. Mohammed Aljaberi, N. Khalid Ansari, Y. Sun, S. Yin, L. Nasifu, H. Sun, T. Xu, Y. Pan and Z. Nie (2023). "Phenotype and genotype analysis for Helicobacter pylori antibiotic resistance in outpatients: a retrospective study." Microbiology Spectrum **11**(5): e00550-00523.
Xu, H., J. Yun, R. Li, X. Ma, L. Gou, T. Che and D. Zhang (2022). "Antibiotics resistance prevalence of Helicobacter pylori strains in Northwest China." Infection and Drug Resistance: 5519-5528.

Yu, L., L. Luo, X. Long, X. Liang, Y. Ji, Q. Chen, Y. Song, X. Li, D. Y. Graham and H. Lu (2019). "Susceptibility-guided therapy for Helicobacter pylori infection treatment failures." Therapeutic advances in gastroenterology **12**: 1756284819874922.

Zerbetto De Palma, G., N. Mendiondo, A. Wonaga, L. Viola, D. Ibarra, E. Campitelli, N. Salim, R. Corti, C. Goldman and M. Catalano (2017). "Occurrence of mutations in the antimicrobial target genes related to levofloxacin, clarithromycin, and amoxicillin resistance in Helicobacter pylori isolates from Buenos Aires City." Microbial Drug Resistance **23**(3): 351-358.

Zhang, J., J. Zhong, J. Ding, J. Shi, T. Tang, Q. Liu, H. Huang, L. Dai and N. Yang (2018). "Simultaneous detection of human CYP2C19 polymorphisms and antibiotic resistance of Helicobacter pylori using a personalised diagnosis kit." Journal of Global Antimicrobial Resistance **13**: 174-179.

Zhang, S., X. Wang, M. J. Wise, Y. He, H. Chen, A. Liu, H. Huang, S. Young, C. Y. Tay and B. J. Marshall (2020). "Mutations of Helicobacter pylori RdxA are mainly related to the phylogenetic origin of the strain and not to metronidazole resistance." Journal of Antimicrobial Chemotherapy **75**(11): 3152-3155.

Zhang, W., Q. Chen, X. Liang, W. Liu, S. Xiao, D. Y. Graham and H. Lu (2015). "Bismuth, lansoprazole, amoxicillin and metronidazole or clarithromycin as first-line Helicobacter pylori therapy." Gut **64**(11): 1715-1720.

Zhang, Y., F. Meng, J. Jin, J. Wang, B.-B. Gu, J.-B. Peng and L.-P. Ye (2021). "Ninety-four thousand-case retrospective study on antibacterial drug resistance of Helicobacter pylori." World Journal of Clinical Cases **9**(35): 10838.

Zhang, Y., Y. Wen, Q. Xiao, W. Zheng, G. Long, B. Chen, X. Shu and M. Jiang (2020). "Mutations in the antibiotic target genes related to clarithromycin, metronidazole and levofloxacin resistance in Helicobacter pylori strains from children in China." Infection and drug resistance: 311-322.

Zhang, Y.-X., L.-Y. Zhou, Z.-Q. Song, J.-Z. Zhang, L.-H. He and Y. Ding (2015). "Primary antibiotic resistance of Helicobacter pylori strains isolated from patients with dyspeptic symptoms in Beijing: a prospective serial study." World journal of gastroenterology: WJG **21**(9): 2786.

Zhou, Y., Z. Zhong, S. Hu, J. Wang, Y. Deng, X. Li, X. Chen, X. Li, Y. Tang and X. Li (2022). "A survey of helicobacter pylori antibiotic-resistant genotypes and strain lineages by whole-genome sequencing in China." Antimicrobial Agents and Chemotherapy **66**(6): e02188-02121.

Ziver-Sarp, T., P. Yuksel-Mayda, S. Saribas, S. Demiryas, N. Gareayaghi, S. Ergin, I. Tasci, D. Ozbey, K. Bal and Y. Erzin (2021). "Point Mutations at gyrA and gyrB Genes of Levofloxacin Resistant Helicobacter pylori Strains and Dual Resistance with Clarithromycin." Clinical Laboratory **67**(10).

Zollner-Schwetz, I., E. Leitner, W. Plieschnegger, G. Semlitsch, V. Stepan, L. Reiter, G. Reicht, E. Mörth, J. Pavek and P. Parsché (2016). "Primary resistance of Helicobacter pylori is still low in Southern Austria." International Journal of Medical Microbiology **306**(4): 206-211.
